# Stabilization of the Skeletal Muscle Ryanodine Receptor Ion Channel-FKBP12 Complex by the 1,4-Benzothiazepine Derivative S107

**DOI:** 10.1371/journal.pone.0054208

**Published:** 2013-01-17

**Authors:** Yingwu Mei, Le Xu, Henning F. Kramer, Ginger H. Tomberlin, Claire Townsend, Gerhard Meissner

**Affiliations:** 1 Department of Biochemistry and Biophysics, University of North Carolina School of Medicine, Chapel Hill, North Carolina, United States of America; 2 GlaxoSmithKline Research and Development, Research Triangle Park, North Carolina, United States of America; Cinvestav-IPN, Mexico

## Abstract

Activation of the skeletal muscle ryanodine receptor (RyR1) complex results in the rapid release of Ca^2+^ from the sarcoplasmic reticulum and muscle contraction. Dissociation of the small FK506 binding protein 12 subunit (FKBP12) increases RyR1 activity and impairs muscle function. The 1,4-benzothiazepine derivative JTV519, and the more specific derivative S107 **(**2,3,4,5,-tetrahydro-7-methoxy-4-methyl-1,4-benzothiazepine), are thought to improve skeletal muscle function by stabilizing the RyR1-FKBP12 complex. Here, we report a high degree of nonspecific and specific low affinity [^3^H]S107 binding to SR vesicles. SR vesicles enriched in RyR1 bound ∼48 [^3^H]S107 per RyR1 tetramer with EC_50_ ∼52 µM and Hillslope ∼2. The effects of S107 and FKBP12 on RyR1 were examined under conditions that altered the redox state of RyR1. S107 increased FKBP12 binding to RyR1 in SR vesicles in the presence of reduced glutathione and the NO-donor NOC12, with no effect in the presence of oxidized glutathione. Addition of 0.15 µM FKBP12 to SR vesicles prevented FKBP12 dissociation; however, in the presence of oxidized glutathione and NOC12, FKBP12 dissociation was observed in skeletal muscle homogenates that contained 0.43 µM myoplasmic FKBP12 and was attenuated by S107. In single channel measurements with FKBP12-depleted RyR1s, in the absence and presence of NOC12, S107 augmented the FKBP12-mediated decrease in channel activity. The data suggest that S107 can reverse the harmful effects of redox active species on SR Ca^2+^ release in skeletal muscle by binding to RyR1 low affinity sites.

## Introduction

The ryanodine receptors (RyRs) are ion channels that release Ca^2+^ from the sarcoplasmic reticulum (SR) in response to an action potential in skeletal and cardiac muscle. RyRs are 2,200 kDa multi-protein complexes composed of four 560-kDa RyR subunits, four small FK506 binding protein (FKBP) subunits and additional associated proteins [1]–[4]. RyRs are regulated by multiple endogenous effectors that include Ca^2+^, Mg^2+^, ATP, protein kinases and redox active species [1]–[4].

FKBP12 associates predominantly with the skeletal muscle isoform to regulate RyR1 function. FKBP12.6 binds with higher affinity to the cardiac muscle isoform RyR2 to modulate cardiac muscle SR Ca^2+^ release [5]. FKBPs belong to a family of immunophilins that exhibit cis/trans isomerase activity. Pharmacological removal of FKBPs using rapamycin or FK506, causes the dissociation of FKBP12 and FKBP12.6 from the RyR macromolecular complexes, uncouples RyR ion channels from their neighbors and activates Ca^2+^ release from SR [6]–[9]. In lipid bilayers, rapamycin and FK506 increase channel activity and lead to the formation of channel openings with reduced conductance which are referred to as substates [6], [7]. Two studies reported that elimination of FKBP12 binding to RyR1 decreased voltage-gated SR Ca^2+^ release in myotubes [10] and fast skeletal muscle fibers [11].

RyR1 and RyR2 channels are subject to post-translational modifications in skeletal and cardiac muscle. In failing hearts, PKA-mediated hyperphosphorylation removed FKBP12.6 from RyR2 and increased channel activity [12]. In a mouse skeletal muscle model of heart failure and in patients with heart disease, exercise was linked to hyperphosphorylation and depletion of FKBP12 from RyR1 that results in increased channel activity and decreased exercise capacity [13], [14]. These findings suggested that hyperphosphorylation and dissociation of the small FKBP subunits (also referred to as calstabins) from RyRs result in leaky SR Ca^2+^ channels and impaired muscle function [15]. However, other laboratories failed to support this proposal [16]–[19]. In addition to PKA-mediated phosphorylation, mechanisms implicated in the generation of leaky Ca^2+^ release channels include oxidation and S-nitrosylation, and dissociation of FKBPs from RyR1 and RyR2. In dystrophic muscle, increased S-nitrosylation of RyR1 [20] and RyR2 [21] resulted in partial dissociation of FKBPs, and the formation of leaky Ca^2+^ channels. Gonzalez et al. [22] reported that elimination of neuronal nitric oxide synthase (nNOS), which is closely associated with RyR2 in cardiac muscle, increased diastolic Ca^2+^ levels. nNOS elimination was associated with decreased S-nitrosylation, increased oxidation of RyR2, leakage of SR Ca^2+^, and arrhythmogenesis in cardiomyocytes. FKBP12.6 binding to RyR2 and RyR2 phosphorylation were not altered in homozygous nNOS knockout mice.

JTV519, a 1,4-benzothiazepine, also known as K201, and the more specific 2,3,4,5,-tetrahydro-7-methoxy-4-methyl-1,4-benzothiazepine, S107, were reported to improve muscle function by stabilizing RyR-FKBP complexes. In an early study, JTV519 reduced SR Ca^2+^ efflux and improved cardiac function in dogs subjected to chronic right ventricular pacing by minimizing RyR2 phosphorylation and stabilizing the RyR2-FKBP12.6 complex [23]. In single channel recordings, JTV519 stabilized the closed state of RyR2 by promoting FKBP12.6 binding [24]. On the other hand, JTV519 suppressed spontaneous Ca^2+^ release in the absence of FKPB12.6 and inhibited [^3^H]ryanodine binding to the RyR2-N4104K mutant that is linked to ventricular tachycardia [25].

In recent studies, S107, the RyR-specific derivative of JTV519, enhanced binding of FKBP12.6 to catecholaminergic polymorphic ventricular tachycardia (CPVT)-linked RyR2-R2474S mutant [26], and FKBP12 to the oxidized and hypernitrosylated RyR1 [20], [27], [28]. Treatment with S107 partially inhibited PKA- and H_2_O_2_-mediated release of FKBP12.6 from cardiac SR vesicles [29] and stabilized the skeletal muscle FKBP12-RyR1 complex in 24-month old mice [28].

In the present study, [^3^H]S107 binding and regulation of RyR1 by FKBP12 and S107 were examined. The results indicate a low equilibrium binding affinity of S107 to multiple RyR1 sites, and provide insight in how FKBP12 and S107 regulate RyR1 under reducing and oxidizing conditions.

## Materials and Methods

### Ethics Statement

This study was carried out in accordance with the recommendations in the Guide for the Care and Use of Laboratory Animals of the National Institutes of Health. The protocol was approved by the University of North Carolina at Chapel Hill Institutional Animal Care and Use Committee (10-056).

### Materials

S107 (HCl salt) was synthesized as described [30]. [^3^H]S107 was prepared by RC TRITEC AG (Teufen, Switzerland). [^3^H]Ryanodine was obtained from Perkin Elmer (Boston, MA), and N-ethyl-2-(1-ethyl-2-hydroxy-2-nitrosohydrazino)ethanamine (NOC12) from Calbiochem Life Sciences (La Jolla, CA). Protease inhibitor cocktail was from Sigma (St Louis, MO) and FK506 from Cayman (Ann Arbor, MI). FKBP12 monoclonal antibody was from R&D Systems (Minneapolis, MN), anti-Cys-SNO polyclonal from Sigma. Anti-RyR1 polyclonal antibody #6425 was prepared by ΨProSci Inc. (Poway, CA). Advanced ECL detection reagent kit was from Amersham Biosciences (Piscataway, NJ). Other chemicals were analytical grade.

### Preparation of Skeletal Muscle Homogenates and Membrane Fractions

Whole muscle homogenates and SR membrane fractions were obtained from leg and back muscle of 2–3 month old rabbits [31]. Rabbit muscle was homogenized at 4°C using a Waring Blender in 6.5 volumes of 0.1 M NaCl, 2 mM EDTA 0.2 mM ethylene glycol-bis(β-aminoethyl ether)-N,N,N’,N’-tetraacetic acid (EGTA), 5 mM Tris maleate, pH 6.8, and protease inhibitors. A crude membrane fraction was obtained by differential centrifugation at 4,500 rpm for 25 min using a Beckman RC3B rotor and centrifugation of the resulting supernatant fraction at 30,000 rpm for 20 min in Ti45 Beckman rotor. After treatment with 0.5 M KCl, membranes were placed on a 20–40% sucrose gradient containing 0.5 M KCl and centrifuged for 16 h at 24,000 rpm in a SW28 Beckman rotor. SR vesicles with a relatively high RyR1 content (Table 1) were recovered at 33–39% sucrose. SR vesicles with low RyR1 content (Table 1) were isolated in the absence of salt. Rabbit muscle was homogenized at 4°C using a Waring Blender in 6.5 volumes of 0.3 M sucrose and protease inhibitors. A crude membrane fraction obtained by differential centrifugation as described above was placed on a 20–40% sucrose gradient. After centrifugation for 16 h at 24,000 rpm in SW28 Beckman rotor, SR vesicles were recovered at 17–24% sucrose. Both gradient fractions were diluted with 2 volumes of H_2_O. After sedimentation by centrifugation, vesicles were suspended in 0.3 M sucrose, 10 mM KPipes, pH 7 at ∼ 10 mg protein/ml. Aliquots of vesicles were quick frozen and stored at −80°C before use. With the exception of Table 1, all experiments were performed with SR vesicles recovered at 33–39% sucrose of gradients.

**Table 1:** [^3^H]S107 binding to SR vesicles.

	[Ca^2+^](µM)	B_max_ of [^3^H]ryanodine binding(pmol/mg protein)	Bound [^3^H]S107(pmol/mg protein)
+FKBP12	50	4.86±0.08	146.0±3.9
+FKBP12	50	0.55±0.05	36.7±8.8
−FKBP12	50	5.10±0.12	181.0±20.4
+FKBP12	<0.01	4.86±0.08	150.4±16.8

SR vesicles with low and high B_max_ of [^3^H]ryanodine binding were obtained as described in Materials and Methods. B_max_ of [^3^H]ryanodine binding was obtained as shown in Fig. S2A. Shown are the amounts of [^3^H]ryanodine specifically bound to SR vesicles after 5 h at 24°C. Specific [^3^H]S107 binding to SR vesicles was determined at 44 µM [^3^H]S107 and indicated free Ca^2+^ concentration as described in Fig. S2B. Shown are the amounts of S107 specifically bound to SR vesicles after 9 h incubation at 24°C. Data are the mean ± SD of 3–6 determinations.

Endogenous FKBP12 was removed by treating SR vesicles for 30 min at 30°C with 10 µM FK506 in 0.3 M sucrose, 0.15 M KCl, 20 mM imidazole, pH 7.5, followed by centrifugation through a layer of 0.5 M sucrose to remove FK506 and the dissociated FKBP12. Removal of FKBP12 was confirmed by immunoblot analysis. Similar results were obtained by incubating vesicles with 10 µM FK506 for 30 min at 37°C in absence of KCl as described previously [32]. Aliquots were quick frozen and stored at −80°C.

### Preparation of Supernatant Fraction

Rabbit skeletal muscle was homogenized at 4°C in 3 volumes of 0.1 M NaCl, 20 mM imidazole, pH 7, and protease inhibitors using a Tekmar Tissumizer for 3×7 s at 13,500 rpm. A supernatant fraction was prepared by centrifuging homogenates for 45 min at 35,000 rpm in a Beckman Ti50 rotor.

### FKBP12 Preparation

Recombinant rabbit FKBP12 engineered with an amino-terminal GST tag and TEV protease cleavage site was purified from E. coli using GST and Q-Sepharose chromatography. Following buffer exchange into phosphate buffered saline and cleavage of the GST affinity tag by the addition of histidine-tagged TEV protease, the cleaved FKBP12 protein was passed through nickel and GST-Sepharose columns to remove the histidine-tagged TEV protease and cleaved GST tag.

### [^3^H]S107 Binding

[^3^H]S107 binding to SR vesicles was determined at 24°C using a centrifugation assay. Unless otherwise indicated, SR vesicles were incubated with 0.1 µM [^3^H]S107 and 0.1 to 100 µM unlabelled S107 in 0.25 M KCl, 20 mM imidazole, pH 7.0 containing 50 µM free Ca^2+^ and protease inhibitors, followed by centrifugation in a Beckman Airfuge for 30 min at 90,000×*g*. Nonspecific binding was determined by measuring [^3^H]S107 binding to SR vesicles incubated for 10 min at 95°C. Bound [^3^H]S107 was determined by scintillation counting after solubilisation of pellets in 50 mM Tris-HCl, pH 8.5 containing 2% SDS. Specific binding parameters were calculated using the four parameter logistic equation (SigmaPlot11).
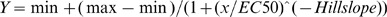
where Y is specifically bound S107 per mg protein, min and max are the minimal and maximal S107 binding values per mg protein, respectively, x is the free S107 concentration, EC50 is the concentration of S107 that results in half-maximal binding, and the Hillslope represents an apparent n_H_.

### Distribution Coefficient of [^3^H]S107

The differential solubility of 0.1 µM [^3^H]S107 between an aqueous and hydrophobic phase was measured using equal volumes 0.25 M KCl, 50 µM Ca^2+^, 20 mM imidazole, pH 7.0 solution and vegetable oil. The two phases were mixed vigorously and separated by centrifugation. Measurement of ^3^H radioactivity in the two phases by liquid scintillation counting yielded a distribution coefficient of 0.61±0.05 (n = 2) between the hydrophobic and aqueous phases.

### Single Channel Recordings

Single channel measurements were performed at 24°C in planar lipid bilayers containing 5∶3∶2 ratio of phosphatidylethanolamine, phosphatidylserine, and phosphatidylcholine (25 mg of total phospholipid/ml of *n*-decane) [33]. SR vesicles were added to the cis cytoplasmic side of the bilayer, with the trans SR luminal side defined as ground. Measurements were made using 0.25 M Cs methanesulfonate, 10 mM CsHepes, pH 7.4 on both sides of the bilayer to minimize K^+^ and Cl^−^ channel activities in SR vesicles [34]. Data acquired using test potentials of ±35 mV were sampled at 10 kHz and filtered at 2 kHz. Channel open probability (P_o_) was determined from at least 2 min of recordings for each condition.

### [^3^H]Ryanodine Binding

The plant alkaloid ryanodine is widely used as a highly specific probe of RyR channel activity because it preferentially binds the open channel configuration [35]. In the present study a relatively low concentration of [^3^H]ryanodine was used to detect changes in binding and thus RyR1 activity. SR vesicles were incubated with 3 nM [^3^H]ryanodine at 24°C in 0.25 M KCl, 20 mM imidazole, pH 7.0 with ∼7 µM free Ca^2+^ and protease inhibitors. Nonspecific binding was determined using 1000-fold excess of unlabelled ryanodine. Aliquots of samples were diluted 9-fold with ice-cold water and placed on Whatman GF/B filters saturated with 2% polyethyleneimine. Filters were washed with three 5-ml volumes of ice-cold 0.1 M KCl, 1 mM K-Pipes, pH 7.0. Radioactivity remaining on the filters was determined by liquid scintillation counting to obtain bound [^3^H]ryanodine.

B_max_ values of [^3^H]ryanodine binding were determined by incubating SR vesicles at 24°C with a near saturating concentration of 20 nM [^3^H]ryanodine in 20 mM imidazole, pH 7.0, 0.6 M KCl, 0.15 M sucrose, protease inhibitors, and 0.1 mM Ca^2+^. Nonspecific binding was determined using a 1000 to 2000 fold excess of unlabeled ryanodine.

### SDS-PAGE and Immunoblot Analysis

RyR1 was detected using 3–12% gradient SDS-PAGE and transfer to nitrocellulose membranes. Experiments for detection of S-nitrosocysteines were performed under nonreducing conditions [36]. Membranes were blotted with 2% ECL Advance blocking reagent in 0.5% Tween 20, Tris buffered saline (TBS), pH 7.4 at 24°C for 2 h. Membranes were probed with primary anti-Cys-SNO polyclonal antibody and secondary peroxidase-conjugated anti-rabbit IgG antibody, and with anti-RyR1 polyclonal antibody and peroxidase-conjugated anti-mouse IgG, using the ECL detection method. FKBP12 was detected using 8–20% gradient SDS-PAGE and anti-FKBP12 monoclonal antibody.

### Coimmunoprecipitation of RyR1 and FKBP12

For co-immunoprecipitation experiments, SR membranes were incubated with and without 44 µM S107 and 0.1 M NOC12 at 24°C for 90 min and solubilized at 2 mg protein/ml in 15 mM NaPipes pH 7.2 containing 0.5 M sucrose, 1 M NaCl, 2.5% Triton X-100 and protease inhibitors [16]. Following incubation for 10 min on ice, samples were diluted with equal volumes of ice-cold water, incubated for another 20 min on ice, and centrifuged for 10 min at 13,000 rpm. After dilution to 0.125 M NaCl and 0.3% Triton X-100 and addition of 0.9 mM Ca^2+^, the supernatant fraction was incubated with RyR1 monoclonal antibody D286 (1∶10 dilution) for 20 h at 4°C. Protein G coupled to magnetic beads was added and samples were incubated for 2 h at 4°C. After washing twice with 20 mM NaPipes, pH 7.2 containing 0.125 M NaCl, 0.3% Triton X-100 and protease inhibitors, bound proteins were released with 30 µl 4× SDS buffer at 37°C for 20 min and separated on SDS gels and analyzed on immunoblots as described above.

### Biochemical Assays and Data Analysis

Free Ca^2+^ concentrations were determined by adding the appropriate amounts of Ca^2+^ and EGTA calculated using stability constants and a published computer program [37]. Free Ca^2+^ concentrations were verified with the use of a Ca^2+^ selective electrode. Unless otherwise indicated, differences between samples were analyzed by one-way Anova followed by Tukey t-test; p<0.05 was considered significant.

## Results

### [^3^H]S107 Binding to SR Vesicles

**Figure 1:**
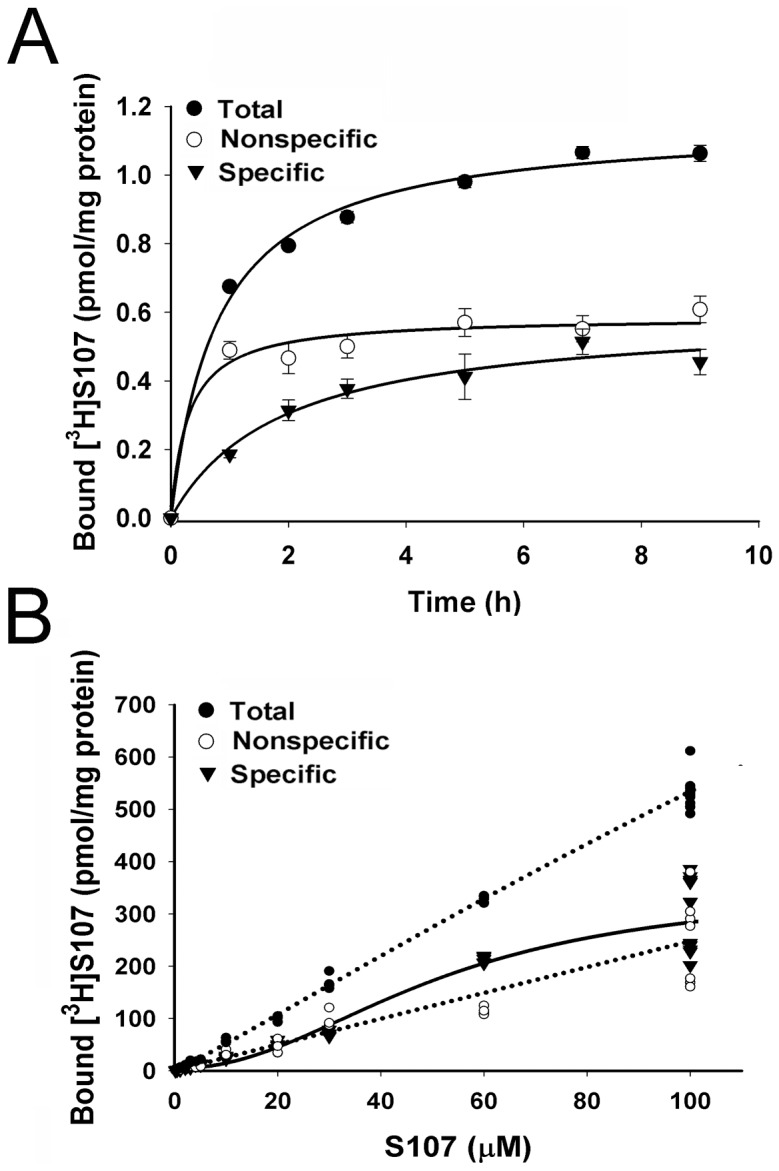
[^3^H]S107 binding to SR vesicles. (A) Time course of total, nonspecific and specific [^3^H]S107 binding to SR vesicles incubated with 0.2 µM [^3^H]S107 in 0.25 M KCl, 20 mM imidazole, pH 7.0, 50 µM free Ca^2+^ and protease inhibitors for 1 to 9 h at 24°C. Nonspecific binding was determined by measuring [^3^H]S107 binding to SR vesicles heat-inactivated for 10 min at 95°C. Data are the mean ± SD of 3 experiments. (B) Total, nonspecific and specific [^3^H]S107 binding to SR vesicles incubated with 0.1 µM [^3^H]S107 and 0.1 to 100 µM S107 as above for 7 h at 24°C. Nonspecific binding was determined as in A. Specific binding curve was obtained using four parameter logistic equation shown in Materials and Methods.

A distribution coefficient of 0.61 (see Materials and Methods) suggests that S107 can be taken up by cells and partition into the large RyR complexes. The time course of total and nonspecific [^3^H]S107 binding was determined by incubating SR vesicles enriched in RyR1 with 0.2 µM [^3^H]S107 for 1 to 9 h at 24°C. Specific [^3^H]S107 binding to the vesicles reached a maximum after ∼7 h (Fig. 1A). A similar time course of specific [^3^H]S107 binding was observed when SR vesicles were incubated with 2 µM [^3^H]S107 and 20 µM [^3^H]S107 (Fig. S1).

Equilibrium binding of [^3^H]S107 was determined in a homologous competition assay. SR vesicles were incubated for 7 h with 0.1 µM [^3^H]S107 in the presence of 0.1 µM to 100 µM unlabelled S107. Total, nonspecific and specific [^3^H]S107 binding are shown in Fig. 1B. Analysis of specific binding using a four parameter logistic equation (see Methods) showed SR vesicles bound 353±46 pmol [^3^H]S107/mg protein (n = 93, p<0.0001) with EC_50_ = 51.8±8.8 µM S107 (p<0.0001) and Hillslope = 2.1±0.4 (p<0.0001). A Hillslope of 2.1 reflected low cooperativity among multiple S107 binding sites. The B_max_ of specific [^3^H]S107 binding exceeded the B_max_ of specific [^3^H]ryanodine binding (7.3±0.3****pmol/mg protein, Fig. S1A), which is a measure of RyR1 content of the vesicles [35].

The high number [^3^H]S107 binding sites (350 sites/7.3 RyR1 or 48 S107 binding sites/RyR1 tetramer) suggested that [^3^H]S107 bound to RyR1 and other SR proteins. We tested this at 44 µM [^3^H]S107. The time course of [^3^H]S107 binding to SR vesicles containing relatively high and low RyR1 concentrations (4.86 and 0.55 pmol [^3^H]ryanodine/mg protein, respectively, Fig. S2) was compared. SR vesicles with low RyR1 content maximally bound 36.7 pmol [^3^H]S107/mg protein after incubation of 1 h (Fig. S2, Table 1). [^3^H]S107 bound more slowly to vesicles with higher RyR1 content, with near maximal binding level of 146 pmol/mg protein after 9 h incubation. Comparison of the binding data suggested that in SR vesicles with high RyR1 content, 80–85% of [^3^H]S107 was bound to RyR1. This corresponded to about 25 [^3^H]S107 molecules per RyR1 tetramer, with the remaining 15–20% bound to other SR components. In SR vesicles with low RyR1 content, ∼38% of [^3^H]S107 was sequestered by RyR1, assuming the binding of 25 [^3^H]S107 per RyR1 at 44 µM [^3^H]S107.

**Figure 2:**
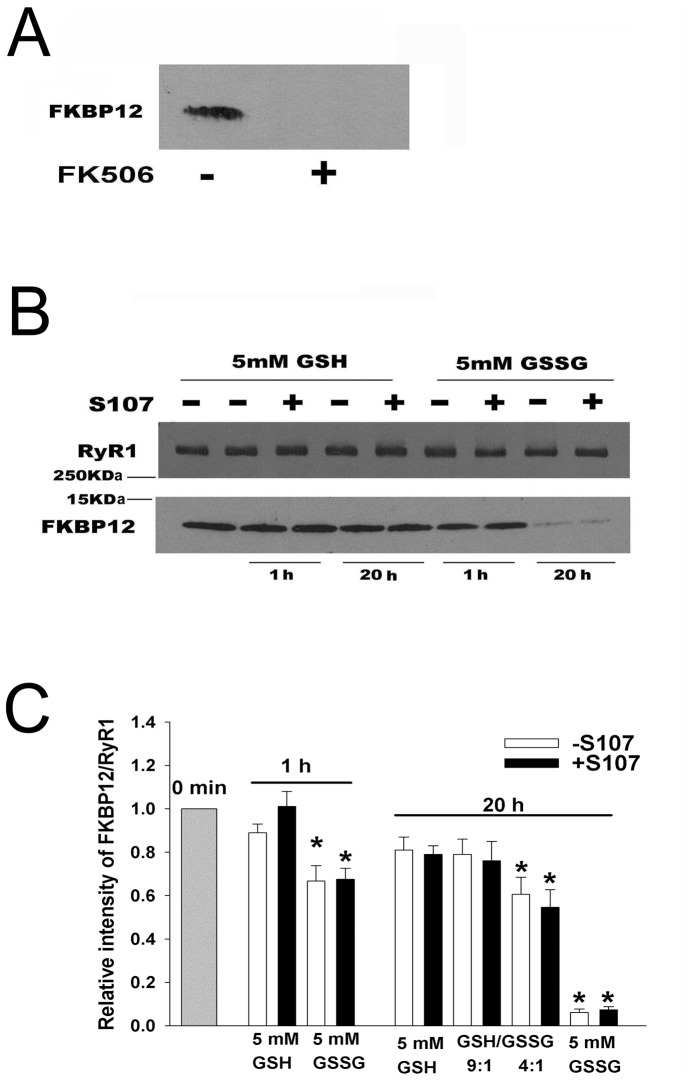
Effects of FK506 and S107 on FKBP12 dissociation from SR vesicles in the presence of GSH and GSSG. (A) Representative immunoblot of SR vesicles not treated and treated with FK506. SR vesicles were incubated with 10 µM FK506 as described in Materials and Methods, followed by centrifugation to remove FK506 and dissociated FKBP12. (B and C) FKBP12 dissociation from SR vesicles in the presence of GSH and GSSG. Immunoblots of SR vesicles not treated with FK506 were incubated for 1 and 20 h at 24°C in 0.25 M KCl, 20 mM imidazole, pH 7.0, 50 µM free Ca^2+^, protease inhibitors, and the indicated concentrations and ratios (5 mM total glutathione) of GSH and GSSG in the absence and presence of 44 µM S107. Free FKBP12 was removed by centrifugation. Data were normalized to SR vesicles not incubated (gray bar, 0 min) and are the mean ± SEM of 4–5 experiments. *p<0.05 compared to SR vesicles at 0 min not treated with S107.

When SR vesicles were treated with FK506 to remove FKBP12 (Fig. 2A), a similar time course of [^3^H]S107 binding was observed with similar amounts of [^3^H]S107 bound to SR vesicles containing or depleted of FKBP12 (Fig. S2, Table 1). A slower time course of [^3^H]S107 binding was observed when vesicles were incubated in the presence of free Ca^2+^ that closed (<0.01 µM Ca^2+^) compared to partially activated (50 µM Ca^2+^) RyR1 (Fig. S2, Table 1). However, similar amounts of [^3^H]S107 were bound to the SR vesicles after incubation of 9 h. We conclude that neither the removal of FKBP12 nor a change in RyR1 activity altered [^3^H]S107 binding to RyR1.

### Effects of S107 on Stability of FKBP12-RyR1 Complex in the Absence of Exogenous FKBP12

RyR1s are redox-sensitive Ca^2+^ channels whose activity is modulated by reduced (GSH) and oxidized (GSSG) glutathione [38]–[40]. To determine the effect of S107 on the stability of the FKBP12-RyR1 complex under reducing and oxidizing conditions, SR vesicles were incubated in the presence of 5 mM GSH or 5 mM GSSG for 1 h and 20 h (Figs. 2B and C). After 1 h in the presence of GSH, 44 µM S107 blocked the release of 10–15% of vesicle-associated FKBP12. Similar amounts (20%) of FKBP12 were released after 20 h in the presence and absence of S107. In the presence of 5 mM GSSG, significantly larger amounts of FKBP12 were released from the vesicles (35% and 95% after incubation for 1 h and 20 h, respectively). Addition of 44 µM S107 did not alter FKBP12 release from RyR1 in the presence of 5 mM GSSG (Figs. 2B and C).

The activity of RyR1 depends on the GSH/GSSG ratio [38], [39]. Incubation of SR vesicles for 20 h with 4.5 mM GSH and 0.5 mM GSSG (9∶1 GSH/GSSG) released amounts of FKBP12 similar to vesicles incubated with 5 mM GSH in the presence and absence of 44 µM S107 (Fig. 2C). About half of FKBP12 content was released from SR vesicles after incubation for 20 h at GSH/GSSG ratio of 4∶1. Taken together, Fig. 2C shows that a decrease in the GSH/GSSG ratio decreased the stability of the FKPB12-RyR1 complex.

**Figure 3:**
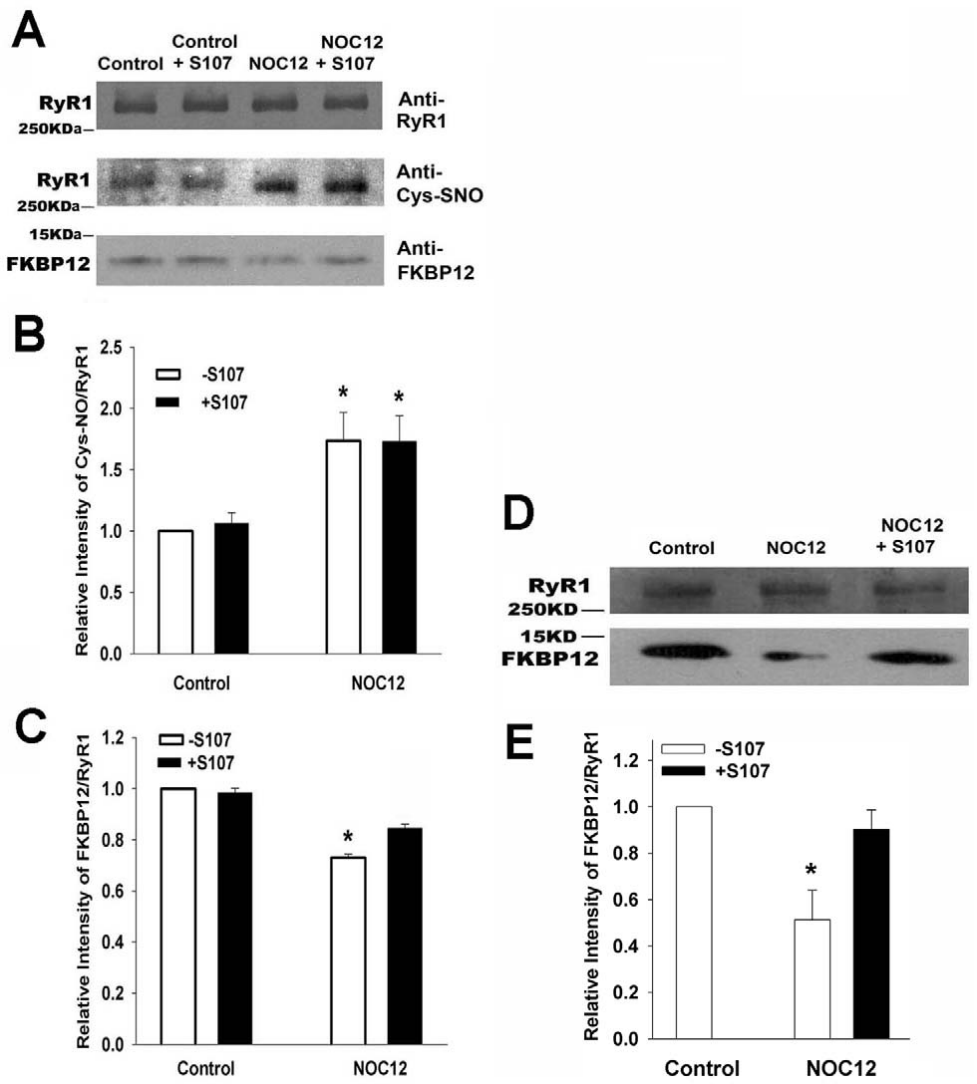
FKBP12 dissociation from SR vesicles in the presence of NOC12. (A–C) SR vesicles not treated with FK506 were incubated for 5 h at 24°C with or without 0.10 mM NOC12 in the absence and presence of 44 µM S107 in 0.25 M KCl, 20 mM imidazole, pH 7.0, 7 µM free Ca^2+^ and protease inhibitors. S-nitrosylation was stopped by centrifugation. Resuspended samples were separated on 8–20% (FKBP12) and 3–12% (RyR1 and Cys-SNO) gradient SDS-PAGE gels and transferred to nitrocellulose membranes to detect S-nitrosylation of RyR1, and FKBP12 and RyR1 proteins. Data are the mean ± SEM of 4 determinations. *p<0.05 compared to control samples (B) and samples with NOC12 and S107 (C). (D and E) SR membranes were incubated with and without 44 µM S107 and 0.1 mM NOC12 at 24°C for 90 min, solubilized, and immunoprecipitated as described in Methods. Immunoblots of RyR1 and FKBP12 are shown. Data are the mean ± SEM of 4 experiments. ^*^p<0.05 compared to control samples and samples incubated with NOC12 and S107.

NO and the NO donor NOC12 activate RyR1 by S-nitrosylation of a single cysteine residue (Cys3635) [36], [41]. Figs. 3A–C show that treatment of SR vesicles with 0.10 mM NOC12 significantly increased RyR1 S-nitrosylation and decreased FKBP12 association with SR vesicles. S107 increased the amount of FKBP12 bound to NOC12-treated vesicles (Fig. 3C) without altering Cys-NO content (Fig. 3B). This suggested that S107 increased the binding affinity of FKBP12 to the S-nitrosylated RyR1.

NOC12- and S107-treated vesicles were solubilized, and RyR1 and FKBP12 co-immunoprecipitated using an antibody against RyR1. Immunoblots indicate that the presence of S107 minimized the dissociation of FKBP12 from the RyR1-FKBP12 complex in SR membranes treated with NOC12 (Figs. 3D and E).

**Figure 4:**
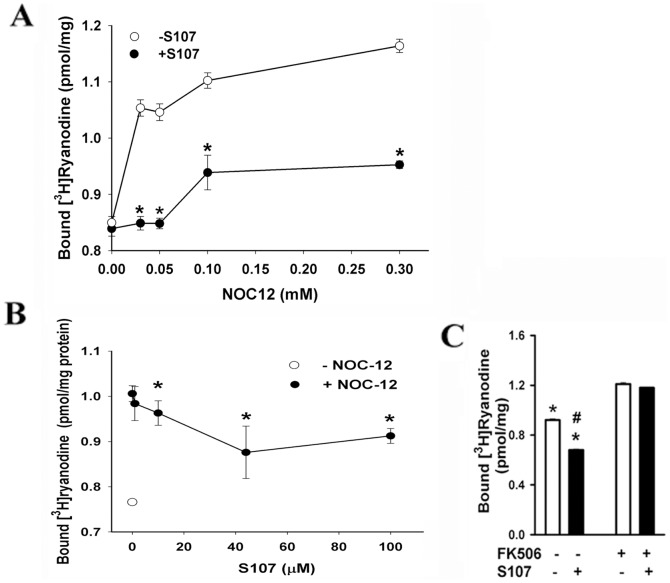
Effects of NOC12 and S107 on [^3^H]ryanodine binding to RyR1. (A) Dependence of [^3^H]ryanodine binding on NOC12 concentration. SR vesicles not treated with FK506 were incubated for 5 h at 24°C in 0.25 M KCl, 20 mM imidazole, pH 7.0, 7 µM free Ca^2+^, protease inhibitors and the indicated concentrations of NOC12 in the presence (•) and absence (○) of 44 µM S107. Data are the mean ± SEM of 4 experiments. *p<0.05 compared to vesicles without S107. (B) Dependence of [^3^H]ryanodine binding to RyR1 on S107 concentration. SR vesicles were incubated as in A in the absence (○) and presence of 50 µM NOC12 (•) and the indicated concentrations of S107. Data are the mean ± SEM of 4 experiments. *p<0.05 compared to vesicles with 50 µM NOC12 and without S107. (C) Specific [^3^H]ryanodine binding to SR vesicles containing (−FK506) and depleted (+FK506) of FKBP12. [^3^H]Ryanodine binding was determined in the presence of 50 µM NOC12 and the absence and presence of 44 µM S107. Data are the mean ± SEM of 8 experiments. *p<0.05 compared to vesicles treated with FK506 and incubated in the absence of S107, ^#^p<0.05 compared to vesicles not treated with FK506 and incubated in the absence of S107.

The effects of NOC12 and S107 on RyR1 activity were further investigated in a ligand binding assay using the RyR1-specific probe ryanodine. Preferential binding [^3^H]ryanodine to the open RyR1 provides an indirect measure of RyR1 activity [35]. NOC12 increased [^3^H]ryanodine binding, suggesting an increase in RyR1 activity at 0.03 to 0.30 mM (Fig. 4A). Activation by NOC12 was partially inhibited by 44 µM S107. More than 10 µM S107 was required to observe a significant decrease in NOC12-induced increase in RyR1 activity using the [^3^H]ryanodine binding assay (Fig. 4B). S107 reduced [^3^H]ryanodine binding to NOC12-treated SR vesicles containing FKBP12 but not depleted of FKBP12 (Fig. 4C). Taken together, the data suggest that S107 decreased NOC12-mediated RyR1 activation by attenuating the dissociation of FKBP12 from RyR1.

### Effect of S107 on the Stability of the FKBP12-RyR1 Complex in Skeletal Muscle Homogenates

SDS-PAGE and immunoblot analysis indicated that the supernatant fraction of rabbit skeletal muscle homogenates contains 0.43±0.05 µM FKBP12 (n = 5) (data not shown). This suggests, considering the 3 volume homogenization buffer, that myoplasmic FKBP12 in rabbit skeletal muscle is ∼1.7 µM, which is in reasonable agreement with a myoplasmic FKBP12 concentration of 3 µM reported previously [42]. To determine the effects of exogenously added and myoplasmic FKBP12 on the stability of the FKBP12-RyR1 complex under reducing and oxidizing conditions, SR vesicles (in presence of 0.15 µM exogenous FKBP12) or homogenates were incubated without or with 5 mM GSH, 5 mM GSSG or 0.1 mM NOC12 for 20 h.

**Figure 5:**
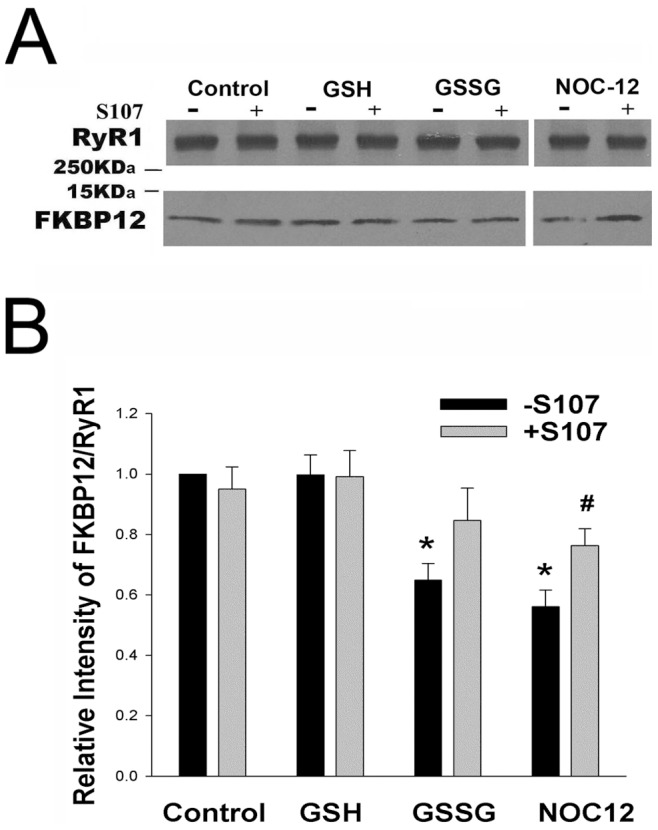
Effect of S107 on the stability of FKBP12-RyR1 complex in skeletal muscle homogenates. (A and B) Skeletal muscle homogenates were incubated without (control) or with 5 mM GSH, 5 mM GSSG or 0.10 mM NOC12 in the absence or presence of 44 µM S107 for 20 h at 24°C. Unbound FKBP12 was removed by centrifugation and the amounts of RyR1 and FKBP12 were detected using anti-RyR1 and anti-FKBP12 antibodies. Homogenates incubated without glutathione and NOC12 served as control. Data are the mean ± SEM of 8 experiments. *p<0.05 compared to control homogenates without S107. ^#^p<0.05 compared to homogenates incubated with NOC12 in the absence of S107.

FKBP12 did not dissociate from SR vesicles incubated with 0.15 µM FKBP12 in the presence of 5 mM GSH, 5 mM GSSG or 0.1 mM NOC12 (Fig. S3). FKBP12 was also not released from the particulate matter of homogenates incubated for 20 h with GSH (Fig. 5). However, in the presence of GSSG and NOC12, ∼35% and 45% FKBP12 dissociation, respectively, was observed in homogenates with 0.43 µM myoplasmic FKBP12. Addition of S107 increased the amount of FKBP12 associated with particulate matter of homogenates, which suggests that S107 minimized GSSG- and NO-mediated dissociation of FKBP12 from RyR1.

### S107 Increases FKBP12 Binding to RyR1

**Figure 6:**
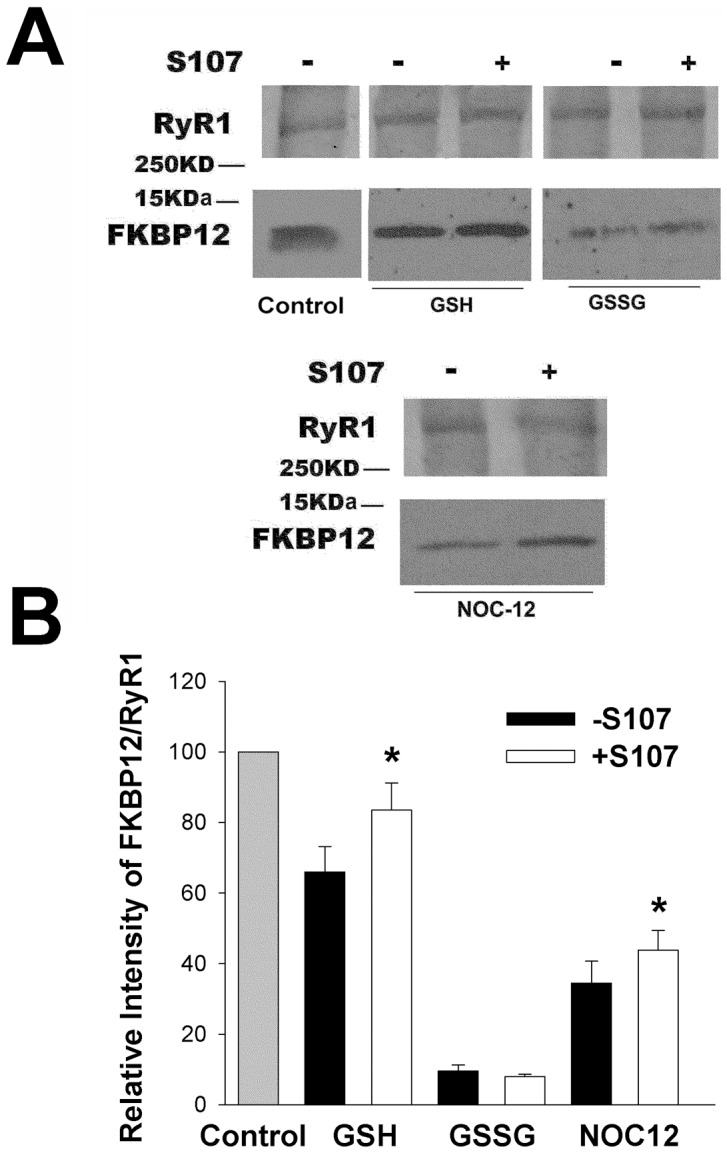
Effects of S107 on FKBP12 binding to RyR1. (A) Immunoblots of RyR1 and FKBP12. SR vesicles depleted of FKBP12 were incubated for 20 h at 24°C in 0.25 M KCl, 20 mM imidazole, pH 7.0, 7 µM free Ca^2+^, protease inhibitors and 10 nM FKBP12 without or with 44 µM S107 in the absence and presence of 5 mM GSH, 5 mM GSSG or 0.10 mM NOC12. SR vesicles not treated with FK506 and incubated without glutathione served as control. (B) SR vesicles not treated with FK506 served as control. Data are the mean of 5–7 experiments. *p<0.05 compared to FKBP12-depleted vesicles incubated without S107 in the presence of GSH and NOC12, respectively, as determined by paired Student’s t-test.

To verify that S107 increases FKBP12 binding to RyR1, FKBP12-depleted SR vesicles were incubated for 20 h with 10 nM FKBP12 in the absence and presence of 44 µM S107 (Fig. 6). In absence of S107, highest levels of FKBP12 binding were seen with SR vesicles incubated in presence of 5 mM GSH, intermediate levels with 0.1 mM NOC12, and lowest levels to SR vesicles with 5 mM GSSG. FKBP12 binding to FKBP12-depleted vesicles was increased by S107 in presence of GSH and NOC12 but not GSSG.

**Figure 7:**
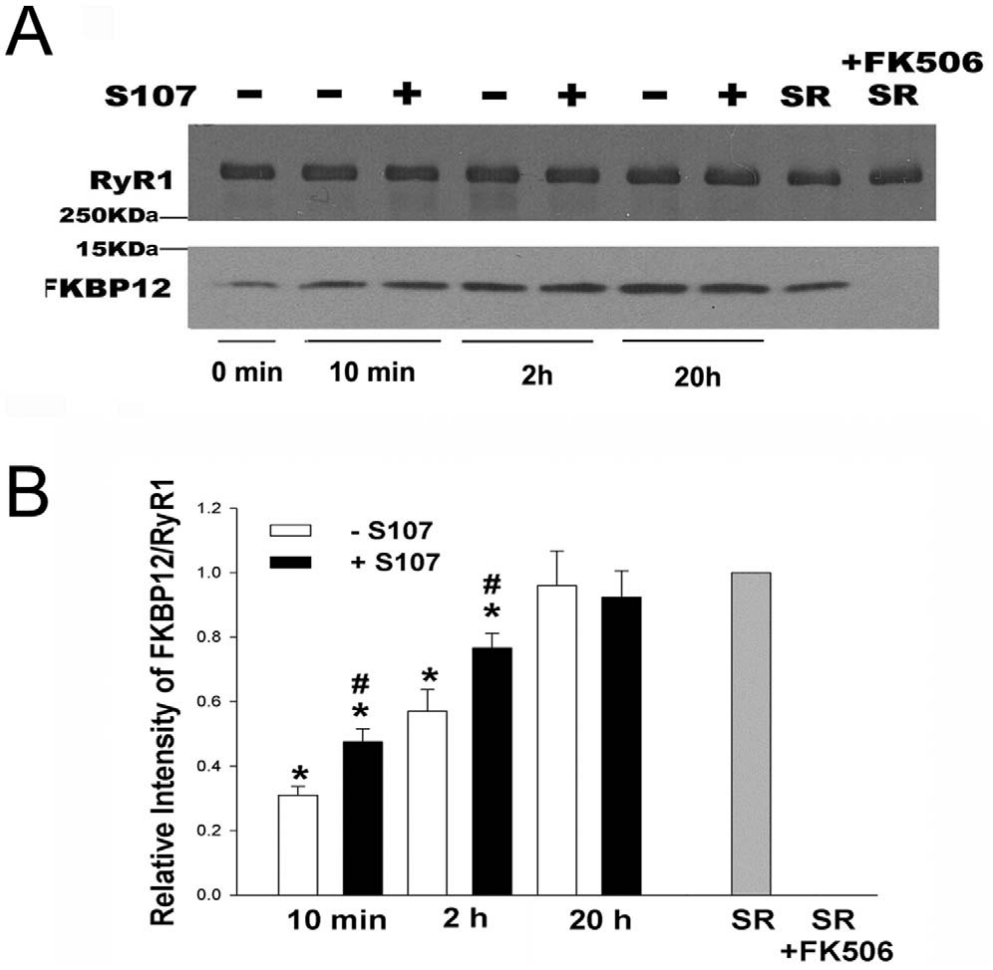
Time course of FKBP12 binding in the presence and absence of S107. (A) Immunoblots of RyR1 and FKBP12. SR vesicles treated with FK506 were incubated in 0.25 M KCl, 20 mM imidazole, pH 7.0, 7 µM free Ca^2+^, protease inhibitors and 1 µM FKBP12 for the indicated times in the presence of 5 mM GSH, and absence or presence of 44 µM S107. FKBP12 binding was stopped by centrifugation. (B) Data are the mean ± SEM of 7 experiments. They were corrected for amounts of FKBP12 associated with FK506 treated SR vesicles kept on ice and normalized to SR vesicles not treated with FK506 (gray bar). *p<0.05 compared to SR vesicles not treated with FK506 and not incubated with FKBP12 and S107. #p<0.05 compared to FKBP12-depleted SR vesicles incubated for the same time (10 min or 2 h) in the presence of FKBP12 but absence of S107.

To determine how S107 affects the association rate of FKBP12 binding to RyR1, FKBP12-depleted SR vesicles were incubated for 10 min, 2 h and 20 h with 1 µM FKBP12 in the presence of 5 mM GSH with and without 44 µM S107, followed by immunoblot analysis (Fig. 7). The amounts of bound FKBP12 were compared with SR vesicles not treated with FK506. After 10 min, significantly greater amounts of FKBP12 were bound to vesicles in the presence than the absence of 44 µM S107 (49±7 and 36±5%, respectively, of FKBP12 in SR vesicles not treated with FK506). Incubation for 2 h significantly increased the level of FKBP12 from 68±4% in the absence of S107 to 86±4% in presence of S107. At 20 h, FKBP12 binding was similar to untreated SR vesicles in the presence and absence of S107. That S107 increased the rate of FKBP12 binding to FKBP12-depleted SR vesicles was confirmed using the [^3^H]ryanodine binding assay (Spplemental Information, Fig. S4).

### Effects of S107 and FKBP12 on RyR1 Single Channel Activity Using the Planar Lipid Bilayer Method

SR vesicles not treated with or exposed to FK506 to remove FKBP12 (Fig. 2A) were fused with a lipid bilayer. Before addition to the cis bilayer chamber, vesicles were incubated for 30 min without and with 25 µM S107 and 5 µM FKBP12, conditions similar to those in Fig. 7. A shorter preincubation period and lower FKBP12 concentration resulted in smaller differences in channel activity without and with S107, whereas longer incubations to achieve equilibrium equalized binding in the absence and presence of S107 (data not shown).

**Figure 8:**
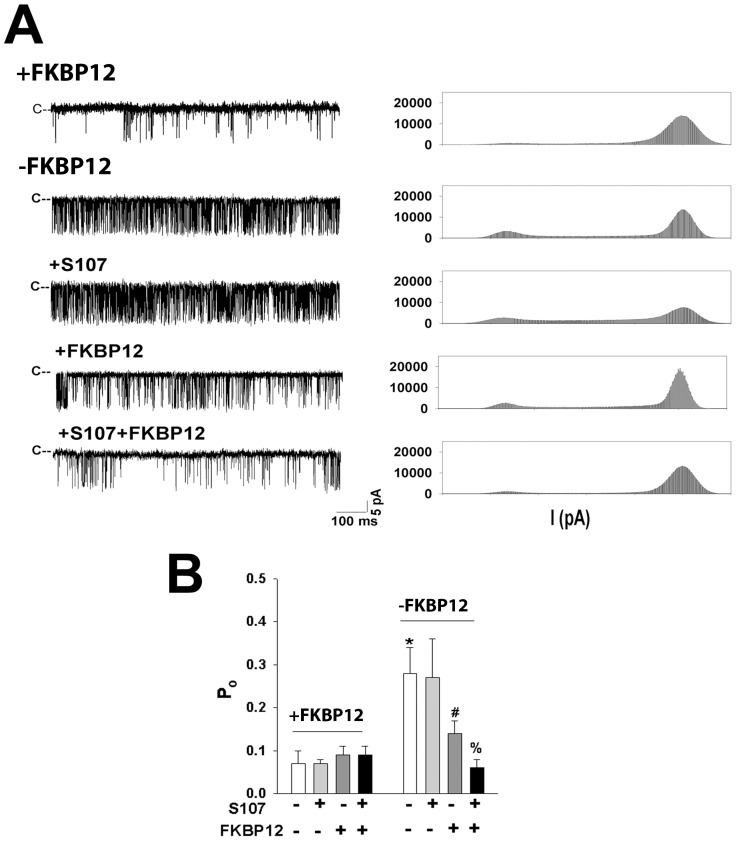
Single channel measurements in the absence and presence of FKBP12 and S107. (A) SR vesicles not treated (top trace) or treated with FK506 (traces 2 and 5) were incubated for 30 min at 24°C without addition (traces 1 and 2), with 25 µM S107 (trace 3), 5 µM FKBP12 (trace 4) or 25 µM S107 plus 5 µM FKBP12 (bottom trace) in 0.3 M sucrose, 0.25 M KCl, 20 mM imidazole, pH 7.0, and protease inhibitors. Vesicles were then fused to a lipid bilayer and recorded at 2 µM cis cytoplasmic Ca^2+^ and −35 mV as described in Materials and Methods. Representative single channel currents (downward deflections from closed levels, c–) (left) and current histograms (right) are shown. (B) Single channel data were obtained as described in A. Data are the mean ± SEM of 4–12 single channel recordings. ^*^p<0.05 compared to RyR1 not treated with FK506 in the absence of S107 and FKBP12. ^#^p<0.05 compared to FK506-treated RyR1 incubated in the absence of S107 and FKBP12. ^%^p<0.05 compared to FK506-treated RyR1 incubated with FKBP12 in absence of S107. p values were determined by Student’s t-test.

FKBP12-depleted RyR1 channels recorded at 2 µM cis cytoplasmic Ca^2+^ exhibited a 4-fold greater single channel open probability (P_o_) than RyR1 channels without FK506 (Fig. 8A, traces 1 and 2, Fig. 8B). Removal of FKBP12 from the RyR1 complex is reported to result in the formation of channel openings with reduced conductance which are referred to as substates [6]. However, current traces and current histograms of Fig. 8A (traces 1 and 2) show that removal of FKBP12 only minimally increased the appearance of substates. A two-fold significant decrease in P_o_ was observed for FKBP12-depleted RyR1 channels preincubated with FKBP12 (Fig. 8A, traces 2 and 4, Fig. 8B). A further 2-fold significant decrease in P_o_ was observed for vesicles preincubated with 5 µM FKBP12 in the presence of 25 µM S107 (Fig. 8A, traces 4 and 5, Fig. 8B). The results suggest that S107 increased binding of FKBP12 to RyR1. In agreement with the [^3^H]ryanodine binding experiments of Fig. 4C, preincubation with 25 µM S107 alone had no effect (Fig. 8A, traces 2 and 3, Fig. 8B). In controls, preincubation with 25 µM S107, 5 µM FKBP12, or the combined preincubation with 25 µM S107 and 5 µM FKBP12 had no effect on channel activities not exposed to FK506 (Fig. 8B). Reduction of cytoplasmic Ca^2+^ from 2 µM to 0.1 µM decreased P_o_ to a similar low value for vesicles treated and not treated with FK506 (data not shown).

Single channel measurements were also performed with SR vesicles preincubated with 0.1 mM NOC12 (Fig. S5). As shown in Fig. 8, S107 lowered P_o_ of FKBP12-depleted RyR1s in the presence but not the absence of FKBP12.

## Discussion

This report describes the regulation of the skeletal muscle RyR1 ion channel by the 1,4 benzoderivative S107 and FKBP12 subunit. Previous studies suggested that S107 reverses the deleterious effects of *in vivo* RyR oxidation and S-nitrosylation on SR Ca^2+^ release by reducing FKBP dissociation from cardiac muscle and skeletal muscle RyR complexes [20], [28], [29]. The present study provides the first direct insights into the S107 binding characteristics of RyR1. [^3^H]S107 binding studies indicate that S107 binds with micromolar affinity to multiple RyR1 sites. Immunoblot, [^3^H]ryanodine binding and single channel measurements showed that S107 inhibited S-nitrosylation-mediated FKBP12 dissociation and RyR1 activation, and attenuated the destabilizing effects of oxidized glutathione and S-nitrosylation in a skeletal muscle homogenate.

[^3^H]S107 binding studies indicated a high degree of nonspecific and specific binding, which suggests that S107 may have additional functional effects by interacting with other SR proteins and possibly RyR1 associated lipids. Analysis of the [^3^H]S107 binding data using a four parameter logistic equation suggested a low binding affinity (EC_50_ ∼50 µM) to RyR1. An unexpectedly high number of binding sites was observed that exceeded the concentration of RyR1 in SR vesicles. Further studies are needed to address why S107 binds slowly to RyR1, how S107 alters the regulation of RyR1 by other endogenous effectors, and the basis of the high number of S107 binding sites in RyR1. A distribution coeffficient of 0.61 suggests that S107 partitions into the large subunits of RyR1 allowing the binding to multiple sites.

Four FKBP12 molecules are bound to the homotetrameric RyRs [32]. Dissociation of FKBP12 from the RyR1 complex increased open channel probability (P_o_) and induced substates [6], [7], [43], [44]. In agreement with Barg at al. [45], we found that removal of FKBP12 increased RyR1 activity at 2 µM free Ca^2+^, but did not increase the frequency of RyR1 substates in single channel measurements. The reason for this discrepancy is unclear but may result from differences in sample preparation and recording conditions.

The RyRs are redox-sensitive channels whose redox state and activity depend on the glutathione redox potential and reactive oxygen and nitrogen species [29], [38], [39], [46]–[50]. There are 100 cysteine residues in the RyR1 subunit and one cysteine per FKBP12 subunit that can be potentially modified [2]. RyR1s are likely in a reduced state *in vivo* based on the reducing environment of thiol-reducing compounds, the most abundant being glutathione [51]. Unlike the RyR1 polypeptides, which are readily modified by redox active species, the small FKBP12 subunit was neither S-nitrosylated using NO-donor NOR-3 nor S-glutathionylated by GSH and H_2_O_2_ [52]. S-nitrosylation decreased RyR1 binding affinity for FKBP12 several fold in a [^35^S]FKBP binding exchange assay, whereas S-glutathionylation had no effect. Treatment with H_2_O_2_ and diamide lowered FKBP12 binding to RyR1 [53]. In the present study with SR vesicles in the absence of exogenously added FKBP12, oxidation resulted in FKBP12 dissociation from the RyR1 complex when free thiol content was decreased from 37.5±1.0 thiols/RyR1 subunit in the presence of GSH to 30.8±2.7 thiols/RyR1 subunit in the presence of GSSG [54], and when one cysteine (Cys3635) per RyR1 subunit was S-nitrosylated by the NO donor NOC12 [36], [41]. The results indicate that oxidation and S-nitrosylation of RyR1 destabilize the FKBP12-RyR1 complex.

S107 has a significant effect on skeletal muscle function by minimizing the release of FKPB12 from RyR1 and thereby NO-mediated SR Ca^2+^ leakage during exercise [30], and oxidation and S-nitrosylation in dystrophic and aged skeletal muscle [20]. Rapamycin-induced deleterious effects on mitochondrial function could be prevented by treatment of FDB fibers with 5 µM S107 [28]. Daily administration of 1.3 mg S107 (∼20 µmol S107/kg) for 4 weeks improved muscle function and exercise capacity in WT but not FKBP12^−/−^ mice [28]. We found that S107 increased FKBP12 binding to RyR1 incubated with the NO donor NOC12. Greater than 10 µM S107 was required to significantly inhibit NOC12-mediated activation of RyR1. This was in agreement with the [^3^H]S107 binding studies, which showed that S107 binds with low affinity to RyR1. Removal of FKBP12 from SR vesicles did not appreciably alter [^3^H]S107 binding, which suggests that S107 binding did not depend on FKBP12.

Previous immunoprecipitation and immunoblot experiments showed that treatment of mice with S107 stabilized binding of FKBP12 to RyR1 without attenuating RyR1 oxidation and S-nitrosylation in skeletal muscle of 24-month-old mice [28]. We observed GSSG- and NO-mediated dissociation of FKBP12 when SR vesicles were incubated in the absence of FKBP12. Presence of 0.15 µM FKBP12 eliminated the dissociation of FKBP12 from SR vesicles. However, GSSG- and NO-mediated dissociation was observed in skeletal muscle homogenates that contained 0.43 µM myoplasmic FKBP12. At this time, the mechanism(s) leading to the dissociation of FKBP12 in homogenates remains unclear. One possibility we cannot rule out is that binding of FKBP12 to myoplasmic proteins decreases the free FKBP12 concentration. In this case, FKBP12 could dissociate from RyR1, and S107 could attenuate dissociation of FKBP12 by increasing FKBP12 binding affinity to RyR1. Another possibility is that unknown factors lead to the release of FKBP12 in homogenates. Further studies will address the mechanism(s) for the release of FKBP12 and enable S107 to stabilize the FKBP12-RyR1 complex in skeletal muscle.

### Conclusion

The present study shows that S107 binds to multiple RyR1 sites with low affinity. Stabilization of the RyR1-FKBP12 complex by S107 depended on the redox state of RyR1. In homogenates, GSSG- and NO-mediated dissociation of FKBP12 occurred in the presence of a relatively high myoplasmic FKBP12 concentration, which suggests that other to be identified factors may be involved in destabilization of the FKBP12-RyR1 complex in skeletal muscle. Reduction of NOC12-mediated release of FKBP12 by S107 in homogenates is of interest, because NO-mediated RyR1 S-nitrosylation triggers the release of Ca^2+^ in brain [55] and skeletal muscle [20], [28], [56] under physiological and pathological conditions.

## Supporting Information

Figure S1 **Time course of specific [^3^H]ryanodine and [^3^H]S107 binding to SR vesicles.** (A) Specific [^3^H]ryanodine binding to SR vesicles was determined as described in Materials and Methods. Data are the mean ± SEM of 6 experiments. (B) SR vesicles were incubated for the indicated times at 24°C with 2 µM (•) and 20 µM (○) [^3^H]S107 in 0.25 M KCl, 20 mM imidazole, pH 7.0, 50 µM free Ca^2+^ and protease inhibitors. Non-specific binding was determined by measuring [^3^H]S107 binding to SR vesicles heat-inactivated for 10 min at 95°C. Data are the mean ± SEM of 4 experiments.
(TIF)

Figure S2 **Time course of specific [^3^Hryanodine and [^3^H]S107 binding to SR vesicles with relatively high and low RyR1 content.** (A) Specific [^3^H]ryanodine binding to SR vesicles with (•,□) and without (Δ) FKBP12 and relatively high and low RyR1 content was determined as described in Materials and Methods. B_max_ of [^3^H]ryanodine binding were 0.55±0.05 (□), 4.86±0.08 (•) and 5.10±0.12 (Δ) pmol/mg protein. Data are the mean ± SD of 3 experiments. (B) SR vesicles with (□,○,•) and without FKBP12 (Δ) were incubated for the indicated times at 24°C with 44 µM [^3^H]S107 in 0.25 M KCl, 20 mM imidazole, pH 7.0, 50 µM (□,•,Δ) and <0.01 µM (○) free Ca^2+^ and protease inhibitors. Nonspecific binding was determined by measuring [^3^H]S107 binding to SR vesicles heat-inactivated for 10 min at 95°C. Data are the mean ± SD of 3–6 experiments.
(JPG)

Figure S3 **Stability of FKBP12-RyR1 complex in presence of 0.15 µM FKBP12.** SR vesicles were incubated in presence of 0.15 µM FKBP12 without (control) and with 5 mM GSH, 5 mM GSSG or 0.10 mM NOC12 for 20 h at 24°C. Unbound FKBP12 was removed by centrifugation and amounts of RyR1 and FKBP12 were detected using anti-RyR1 and anti-FKBP12 antibodies. Data are the mean ± SD of 3 experiments.
(TIF)

Figure S4 **Effects of FKBP12 and S107 on [^3^H]ryanodine binding to SR vesicles not treated and treated with FK506.** (A) Time course of specific [^3^H]ryanodine binding. Vesicles treated and not treated with FK506 were incubated at 24°C for indicated times in 0.25 M KCl, 20 mM imidazole, pH 7.0, 3 nM [^3^H]ryanodine, 7 µM free Ca^2+^, 5 mM GSH and protease inhibitors. Removal of FKBP12 increased RyR1 activity, as indicated by an increase in [^3^H]ryanodine binding. Data are the mean ± SD of 3–4 experiments. *p<0.05 compared to vesicles minus FK506. (B) Effects of FKBP12 and S107 on [^3^H]ryanodine binding. SR vesicles treated and not treated with FK506 were incubated at 24°C for the indicated times in the above buffer containing the indicated concentrations of FKBP12 and S107. Data show that in the absence of added FKBP12, S107 did not alter [^3^H]ryanodine binding to SR vesicles with (−FK506) or freed (+FK506) of FKBP12, whereas S107 significantly decreased [^3^H]ryanodine binding to FKBP12-depleted vesicles in the presence of FKBP12 after preincubation for 10 min and 30 min. Data are the mean ± SEM of 4–6 experiments. *p<0.05 compared to vesicles minus FK506 at 10 and 30 min, respectively.^ #^p<0.05 compared to FKBP12-depleated vesicles plus added FKBP12 and minus S107 at 10 and 30 min, respectively.
(TIF)

Figure S5 **Single channel measurements with NOC12-treated RyR1s.** (A) FK506-treated SR vesicles were incubated for 30 min at 24°C with 0.10 mM NOC12 in 0.3 M sucrose, 0.25 M KCl, 20 mM imidazole, pH 7.0 without (top trace), with 44 µMS107 (trace 2), 5 µM FKBP12 (trace 3) or 44 µM S107 plus 5 µM FKBP12 (bottom trace). Vesicles were then fused to a lipid bilayer and recorded at 2 µM cis cytoplasmic Ca^2+^ and −35 mV as described in Materials and Methods. Representative single channel recordings (downward deflections from closed levels, c–) (left) and current histograms (right) are shown. (B) Single channel data were obtained as described in A. Mean channel open probabilities (Po) were greater in the presence of NOC12 (this figure) than absence of NOC12 (Fig. 8) by 11% in absence of S107 and FKBP12, by 11% in presence of S107, by 3% in presence of FKBP12, and 35% in presence of S107 and FKBP12. Data are the mean ± SEM of 5–8 single channel recordings. ^*^p<0.05 compared to RyR1s not incubated with S107 and FKBP12, ^#^p<0.05 compared to RyR1s incubated with FKBP12 in absence of S107. Significance of differences of data was analyzed with Student’s t-test.
(TIF)

## References

[1] Franzini-Armstrong C, Protasi F (1997) Ryanodine receptors of striated muscles: a complex channel capable of multiple interactions. Physiol Rev 77: 699–729. 9234963 10.1152/physrev.1997.77.3.699

[2] Meissner G (2002) Regulation of mammalian ryanodine receptors. Frontiers Biosci 7: d2072–2080. 10.2741/A899 12438018

[3] Lanner JT, Georgiou DK, Joshi AD, Hamilton SL (2010) Ryanodine receptors: structure, expression, molecular details, and function in calcium release. Cold Spring Harb Perspect Biol 2: a003996. 20961976 10.1101/cshperspect.a003996 PMC2964179

[4] Capes EM, Loaiza R, Valdivia HH (2011) Ryanodine receptors. Skeletal Muscle 1: 18. 21798098 10.1186/2044-5040-1-18 PMC3156641

[5] Lam E, Martin MM, Timerman AP, Sabers C, Fleischer S, et al (1995) A novel FK506 binding protein can mediate the immunosuppressive effects of FK506 and is associated with the cardiac ryanodine receptor. J Biol Chem 270: 26511–26522. 7592869 10.1074/jbc.270.44.26511

[6] Brillantes AB, Ondrias K, Scott A, Kobrinsky E, Ondriasova E, et al (1994) Stabilization of calcium release channel (ryanodine receptor) function by FK506-binding protein. Cell 77: 513–523. 7514503 10.1016/0092-8674(94)90214-3

[7] Ahern GP, Junankar PR, Dulhunty AF (1997) Subconductance states in single-channel activity of skeletal muscle ryanodine receptors after removal of FKBP12. Biophys J 72: 146–162. 8994600 10.1016/S0006-3495(97)78654-5 PMC1184304

[8] Marx SO, Ondrias K, Marks AR (1998) Coupled gating between individual skeletal muscle Ca^2+^ release channels (ryanodine receptors). Science 281: 818–821. 9694652 10.1126/science.281.5378.818

[9] Marx SO, Gaburjakova J, Gaburjakova M, Henrikson C, Ondrias K, et al (2001) Coupled gating between cardiac calcium release channels (ryanodine receptors). Circ Res 88: 1151–1158. 11397781 10.1161/hh1101.091268

[10] Avila G, Lee EH, Perez CF, Allen PD, Dirksen RT (2003) FKBP12 binding to RyR1 modulates excitation-contraction coupling in mouse skeletal myotubes. J Biol Chem 278: 22600–22608. 12704193 10.1074/jbc.M205866200

[11] Tang W, Ingalls CP, Durham WJ, Snider J, Reid MB, et al (2004) Altered excitation-contraction coupling with skeletal muscle specific FKBP12 deficiency. The FASEB J 18: 1597–1599. 15289441 10.1096/fj.04-1587fje

[12] Marx SO, Reiken S, Hisamatsu Y, Jayaraman T, Burkhoff D, et al (2000) PKA phosphorylation dissociates FKBP12.6 from the calcium release channel (ryanodine receptor): defective regulation in failing hearts. Cell 101: 365–376. 10830164 10.1016/s0092-8674(00)80847-8

[13] Reiken S, Lacampagne A, Zhou H, Kherani A, Lehnart SE, et al (2003) PKA phosphorylation activates the calcium release channel (ryanodine receptor) in skeletal muscle: defective regulation in heart failure. J Cell Biol 160: 919–928. 12629052 10.1083/jcb.200211012 PMC2173774

[14] Ward CW, Reiken S, Marks AR, Marty I, Vassort G, et al (2003) Defects in ryanodine receptor calcium release in skeletal muscle from post-myocardial infarct rats. FASEB J 17: 1517–1519. 12824280 10.1096/fj.02-1083fje

[15] Wehrens XH, Lehnart SE, Marks AR (2005) Intracellular calcium release and cardiac disease. Annu Rev Physiol 67: 69–98. 15709953 10.1146/annurev.physiol.67.040403.114521

[16] Stange M, Xu L, Balshaw D, Yamaguchi N, Meissner G (2003) Characterization of recombinant skeletal muscle (Ser-2843) and cardiac muscle (Ser-2809) ryanodine receptor phosphorylation mutants. J Biol Chem 278: 51693–51702. 14532276 10.1074/jbc.M310406200

[17] Xiao B, Jiang MT, Zhao M, Yang D, Sutherland C, et al (2005) Characterization of a novel PKA phosphorylation site, Serine-2030, reveals no PKA hyperphosphorylation of the cardiac ryanodine receptor in canine heart failure. Circ Res 96: 847–855. 15790957 10.1161/01.RES.0000163276.26083.e8

[18] Xiao J, Tian X, Jones PP, Bolstad J, Kong H, et al (2007) Removal of FKBP12.6 does not alter the conductance and activation of the cardiac ryanodine receptor or the susceptibility to stress-induced ventricular arrhythmias. J Biol Chem 282: 34828–34838. 17921453 10.1074/jbc.M707423200 PMC2760432

[19] Li Y, Kranias EG, Mignery GA, Bers DM (2002) Protein kinase A phosphorylation of the ryanodine receptor does not affect calcium sparks in mouse ventricular myocytes. Circ Res 90: 309–316. 11861420 10.1161/hh0302.105660

[20] Bellinger AM, Reiken S, Carlson C, Mongillo M, Liu X, et al (2009) Hypernitrosylated ryanodine receptor calcium release channels are leaky in dystrophic muscle. Nat Med 15: 325–330. 19198614 10.1038/nm.1916 PMC2910579

[21] Fauconnier J, Thireau J, Reiken S, Cassan C, Richard S, et al (2010) Leaky RyR2 trigger ventricular arrhythmias in Duchenne muscular dystrophy. Proc Natl Acad Sci U S A 107: 1559–1564. 20080623 10.1073/pnas.0908540107 PMC2824377

[22] Gonzalez DR, Beigi F, Treuer AV, Hare JM (2007) Deficient ryanodine receptor S-nitrosylation increases sarcoplasmic reticulum calcium leak and arrhythmogenesis in cardiomyocytes. Proc Natl Acad Sci U S A 104: 20612–20617. 18077344 10.1073/pnas.0706796104 PMC2154479

[23] Yano M, Kobayashi S, Kohno M, Doi M, Tokuhisa T, et al (2003) FKBP12.6-mediated stabilization of calcium-release channel (ryanodine receptor) as a novel therapeutic strategy against heart failure. Circulation 107: 477–484. 12551874 10.1161/01.cir.0000044917.74408.be

[24] Wehrens XH, Lehnart SE, Reiken SR, Deng SX, Vest JA, et al (2004) Protection from cardiac arrhythmia through ryanodine receptor-stabilizing protein calstabin 2. Science 304: 292–296. 15073377 10.1126/science.1094301

[25] Hunt DJ, Jones PP, Wang R, Chen W, Bolstad J, et al (2007) K201 (JTV519) suppresses spontaneous Ca^2+^ release and [^3^H]ryanodine binding to RyR2 irrespective of FKBP12.6 association. Biochem J 404: 431–438. 17313373 10.1042/BJ20070135 PMC1896290

[26] Lehnart SE, Mongillo M, Bellinger A, Lindegger N, Chen BX, et al (2008) Leaky Ca^2+^ release channel/ryanodine receptor 2 causes seizures and sudden cardiac death in mice. J Clin Invest 118: 2230–2245. 18483626 10.1172/JCI35346 PMC2381750

[27] Fauconnier J, Meli AC, Thireau J, Roberge S, Shan J, et al (2011) Ryanodine receptor leak mediated by caspase-8 activation leads to left ventricular injury after myocardial ischemia-reperfusion. Proc Natl Acad Sci U S A 108: 13258–13263. 21788490 10.1073/pnas.1100286108 PMC3156220

[28] Andersson DC, Betzenhauser MJ, Reiken S, Meli AC, Umanskaya A, et al (2011) Ryanodine receptor oxidation causes intracellular calcium leak and muscle weakness in aging. Cell Metabolism 14: 196–207. 21803290 10.1016/j.cmet.2011.05.014 PMC3690519

[29] Shan J, Betzenhauser MJ, Kushnir A, Reiken S, Meli AC, et al (2010) Role of chronic ryanodine receptor phosphorylation in heart failure and beta-adrenergic receptor blockade in mice. J Clin Invest 120: 4375–4387. 21099115 10.1172/JCI37649 PMC2993577

[30] Bellinger AM, Reiken S, Dura M, Murphy PW, Deng SX, et al (2008) Remodeling of ryanodine receptor complex causes “leaky” channels: a molecular mechanism for decreased exercise capacity. Proc Natl Acad Sci U S A 105: 2198–2202. 18268335 10.1073/pnas.0711074105 PMC2538898

[31] Anderson K, Cohn AH, Meissner G (1994) High-affinity [^3^H]PN200-110 and [^3^H]ryanodine binding to rabbit and frog skeletal muscle. Am J Physiol 266: C462–C466. 8141261 10.1152/ajpcell.1994.266.2.C462

[32] Timerman AP, Ogunbumni E, Freund E, Wiederrecht G, Marks AR, et al (1993) The calcium release channel of sarcoplasmic reticulum is modulated by FK-506-binding protein. Dissociation and reconstitution of FKBP-12 to the calcium release channel of skeletal muscle sarcoplasmic reticulum. J Biol Chem 268: 22992–22999. 7693682

[33] Xu L, Tripathy A, Pasek DA, Meissner G (1999) Ruthenium red modifies the cardiac and skeletal muscle Ca^2+^ release channels (ryanodine receptors) by multiple mechanisms. J Biol Chem 274: 32680–32691. 10551824 10.1074/jbc.274.46.32680

[34] Meissner G (1983) Monovalent ion and calcium ion fluxes in sarcoplasmic reticulum. Mol Cell Biochem 55: 65–82. 6312285 10.1007/BF00229243

[35] Sutko J L, Airey JA, Welch W, Ruest L (1997) The pharmacology of ryanodine and related compounds. Pharmacol Rev 49: 53–98. 9085309

[36] Sun J, Xin C, Eu JP, Stamler JS, Meissner G (2001) Cysteine-3635 is responsible for skeletal muscle ryanodine receptor modulation by NO. Proc Natl Acad Sci U S A. 98: 11158–11162. 10.1073/pnas.201289098 PMC58700 11562475

[37] Schoenmakers TJ, Visser GJ, Flik G, Theuvenet AP (1992) CHELATOR: an improved method for computing metal ion concentrations in physiological solutions. BioTechniques 12: 870–879. 1642895

[38] Zable AC, Favero TG, Abramson JJ (1997) Glutathione modulates ryanodine receptor from skeletal muscle sarcoplasmic reticulum. Evidence for redox regulation of the Ca^2+^ release mechanism. J Biol Chem 272: 7069–7077. 9054399 10.1074/jbc.272.11.7069

[39] Feng W, Liu G, Allen PD, Pessah IN (2000) Transmembrane redox sensor of ryanodine receptor complex. J Biol Chem 275: 35902–35907. 10998414 10.1074/jbc.C000523200

[40] Balshaw DM, Xu L, Yamaguchi N, Pasek DA, Meissner G (2001) Calmodulin binding and inhibition of cardiac muscle calcium release channel (ryanodine receptor). J Biol Chem 276: 20144–20153. 11274202 10.1074/jbc.M010771200

[41] Sun J, Xu L, Eu JP, Stamler JS, Meissner G (2003) Nitric oxide, NOC-12, and S-nitrosoglutathione modulate the skeletal muscle calcium release channel/ryanodine receptor by different mechanisms. J Biol Chem 278: 8184–8189. 12509428 10.1074/jbc.M211940200

[42] Timerman AP, Wiederrecht G, Marcy A, Fleischer S (1995) Characterization of an exchange reaction between soluble FKBP-12 and the FKBP-ryanodine receptor complex. Modulation by FKBP mutants deficient in peptidyl-prolyl isomerase activity. J Biol Chem 270: 2451–2459. 7531689 10.1074/jbc.270.6.2451

[43] Ma J, Bhat MB, Zhao J (1995) Rectification of skeletal muscle ryanodine receptor mediated by FK506 binding protein. Biophys J 69: 2398–2404. 8599646 10.1016/S0006-3495(95)80109-8 PMC1236477

[44] Kaftan E, Marks AR, Ehrlich BE (1996) Effects of rapamycin on ryanodine receptor/Ca^2+^-release channels from cardiac muscle. Circ Res 78: 990–997. 8635249 10.1161/01.res.78.6.990

[45] Barg S, Copello JA, Fleischer S (1997) Different interactions of cardiac and skeletal muscle ryanodine receptors with FK-506 binding protein isoforms. Am J Physiol 272: C1726–C1733. 9176165 10.1152/ajpcell.1997.272.5.C1726

[46] Reid MB (1996) Reactive oxygen and nitric oxide in skeletal muscle. News Physiol Science 11: 114–121.

[47] Terentyev D, Gyorke I, Belevych AE, Terentyeva R, Sridhar A, et al (2008) Redox modification of ryanodine receptors contributes to sarcoplasmic reticulum Ca^2+^ leak in chronic heart failure. Circ Res 103: 1466–1472. 19008475 10.1161/CIRCRESAHA.108.184457 PMC3274754

[48] Eu JP, Sun J, Xu L, Stamler JS, Meissner G (2000) The skeletal muscle calcium release channel: coupled O_2_ sensor and NO signaling functions. Cell 102: 499–509. 10966111 10.1016/s0092-8674(00)00054-4

[49] Xia R, Stangler T, Abramson JJ (2000) Skeletal muscle ryanodine receptor is a redox sensor with a well defined redox potential that is sensitive to channel modulators. J Biol Chem 275: 36556–36561. 10952995 10.1074/jbc.M007613200

[50] Hidalgo C, Donoso P (2008) Crosstalk between calcium and redox signaling: from molecular mechanisms to health implications. Antioxid Redox Signal 10: 1275–1312. 18377233 10.1089/ars.2007.1886

[51] Deneke SM, Fanburg BL (1989) Regulation of cellular glutathione. Am J Physiol 257: L163–L173. 2572174 10.1152/ajplung.1989.257.4.L163

[52] Aracena P, Tang W, Hamilton SL, Hidalgo C (2005) Effects of S-glutathionylation and S-nitrosylation on calmodulin binding to triads and FKBP12 binding to type 1 calcium release channels. Antioxid Redox Signal 7: 870–881. 15998242 10.1089/ars.2005.7.870

[53] Zissimopoulos S, Docrat N, Lai FA (2007) Redox sensitivity of the ryanodine receptor interaction with FK506-binding protein. J Biol Chem 282: 6976–6983. 17200109 10.1074/jbc.M607590200

[54] Petrotchenko EV, Yamaguchi N, Pasek DA, Borchers CH, Meissner G (2011) Mass spectrometric analysis and mutagenesis predict involvement of multiple cysteines in redox regulation of the skeletal muscle ryanodine receptor ion channel complex. Res Rep Biol 2011: 13–21. 21603587 10.2147/RRB.S15776 PMC3095966

[55] Kakizawa S, Yamazawa T, Chen Y, Ito A, Murayama T, et al (2011) Nitric oxide-induced calcium release via ryanodine receptors regulates neuronal function. EMBO J 31: 417–428. 22036948 10.1038/emboj.2011.386 PMC3261553

[56] Eu JP, Hare JM, Hess DT, Skaf M, Sun J, et al (2003) Concerted regulation of skeletal muscle contractility by oxygen tension and endogenous nitric oxide. Proc Natl Acad Sci U S A 100: 15229–15234. 14645704 10.1073/pnas.2433468100 PMC299968

