# Supplementary figures and images for: Stabilization of the Skeletal Muscle Ryanodine Receptor Ion Channel-FKBP12 Complex by the 1,4-Benzothiazepine Derivative S107
Source: PLoS One. 2013 Jan 17;8(1):e54208. doi: 10.1371/journal.pone.0054208 (PMC3547879; doi:10.1371/journal.pone.0054208)

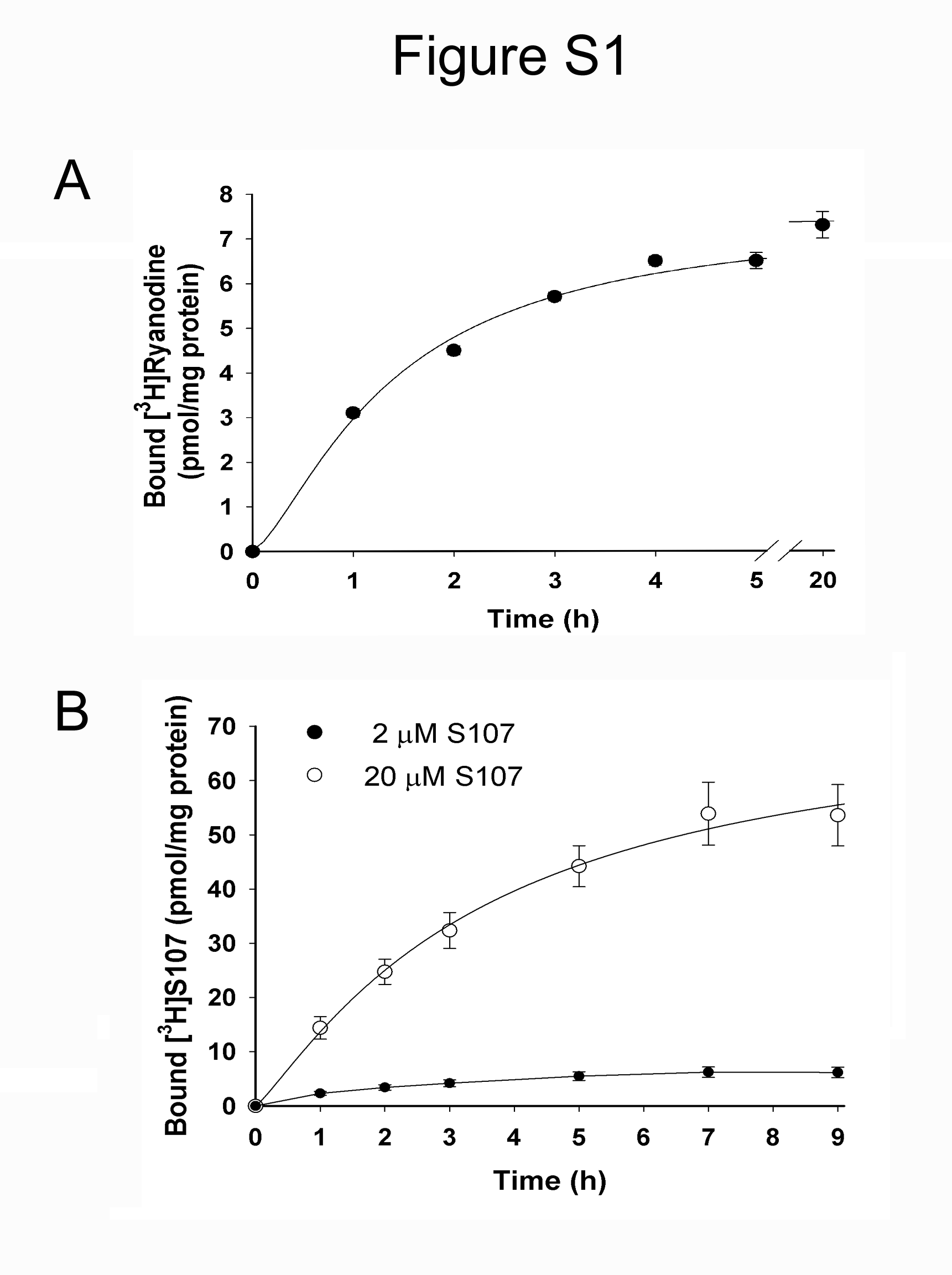

Supplement: Figure S1 — Time course of specific [3H]ryanodine and [3H]S107 binding to SR vesicles. (A) Specific [3H]ryanodine binding to SR vesicles was determined as described in Materials and Methods. Data are the mean ± SEM of 6 experiments. (B) SR vesicles were incubated for the indicated times at 24°C with 2 µM (•) and 20 µM (○) [3H]S107 in 0.25 M KCl, 20 mM imidazole, pH 7.0, 50 µM free Ca2+ and protease inhibitors. Non-specific binding was determined by measuring [3H]S107 binding to SR vesicles heat-inactivated for 10 min at 95°C. Data are the mean ± SEM of 4 experiments. (TIF) [file pone.0054208.s001.tif]

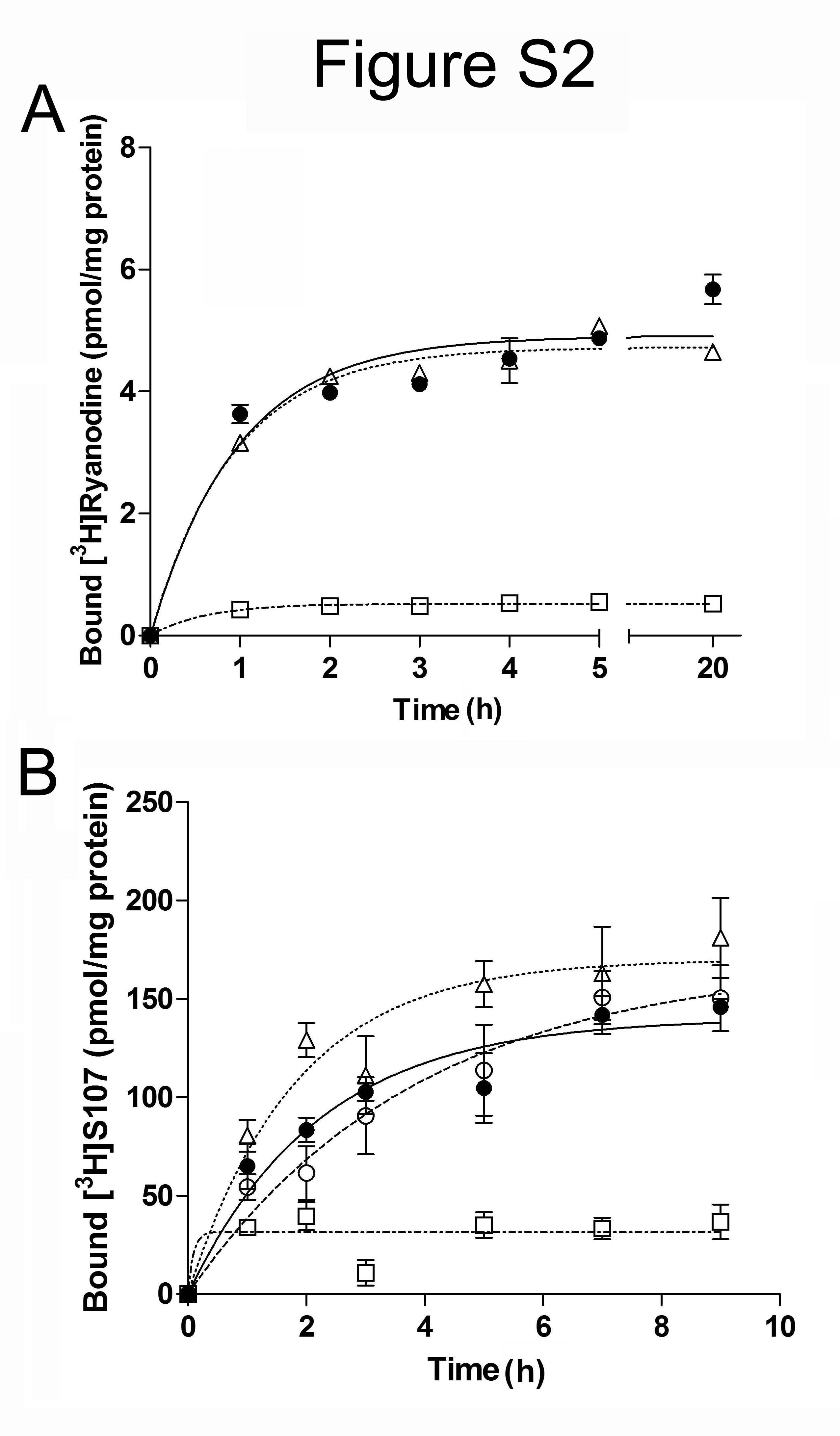

Supplement: Figure S2 — Time course of specific [3Hryanodine and [3H]S107 binding to SR vesicles with relatively high and low RyR1 content. (A) Specific [3H]ryanodine binding to SR vesicles with (•,□) and without (Δ) FKBP12 and relatively high and low RyR1 content was determined as described in Materials and Methods. Bmax of [3H]ryanodine binding were 0.55±0.05 (□), 4.86±0.08 (•) and 5.10±0.12 (Δ) pmol/mg protein. Data are the mean ± SD of 3 experiments. (B) SR vesicles with (□,○,•) and without FKBP12 (Δ) were incubated for the indicated times at 24°C with 44 µM [3H]S107 in 0.25 M KCl, 20 mM imidazole, pH 7.0, 50 µM (□,•,Δ) and <0.01 µM (○) free Ca2+ and protease inhibitors. Nonspecific binding was determined by measuring [3H]S107 binding to SR vesicles heat-inactivated for 10 min at 95°C. Data are the mean ± SD of 3–6 experiments. (JPG) [file pone.0054208.s002.jpg]

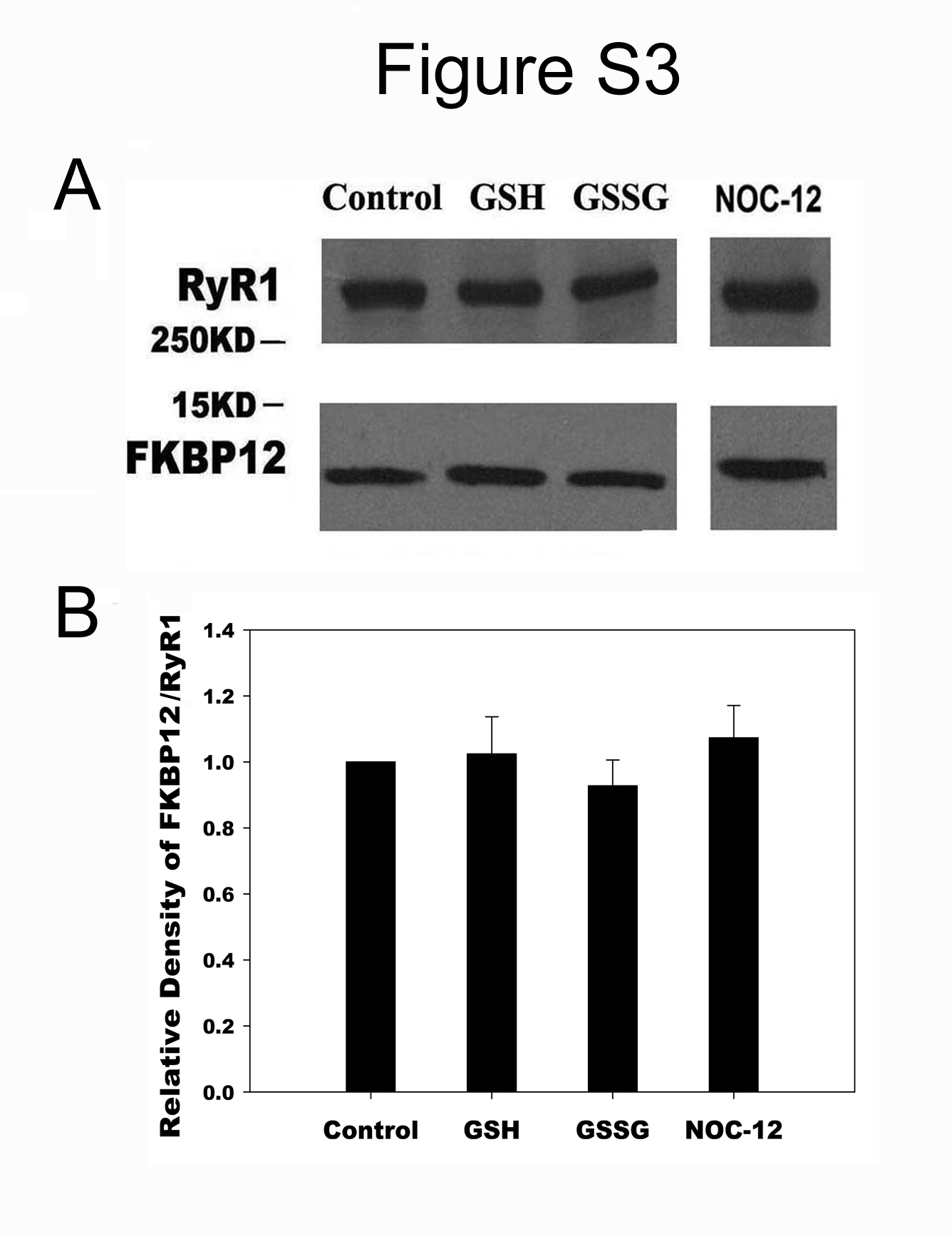

Supplement: Figure S3 — Stability of FKBP12-RyR1 complex in presence of 0.15 µM FKBP12. SR vesicles were incubated in presence of 0.15 µM FKBP12 without (control) and with 5 mM GSH, 5 mM GSSG or 0.10 mM NOC12 for 20 h at 24°C. Unbound FKBP12 was removed by centrifugation and amounts of RyR1 and FKBP12 were detected using anti-RyR1 and anti-FKBP12 antibodies. Data are the mean ± SD of 3 experiments. (TIF) [file pone.0054208.s003.tif]

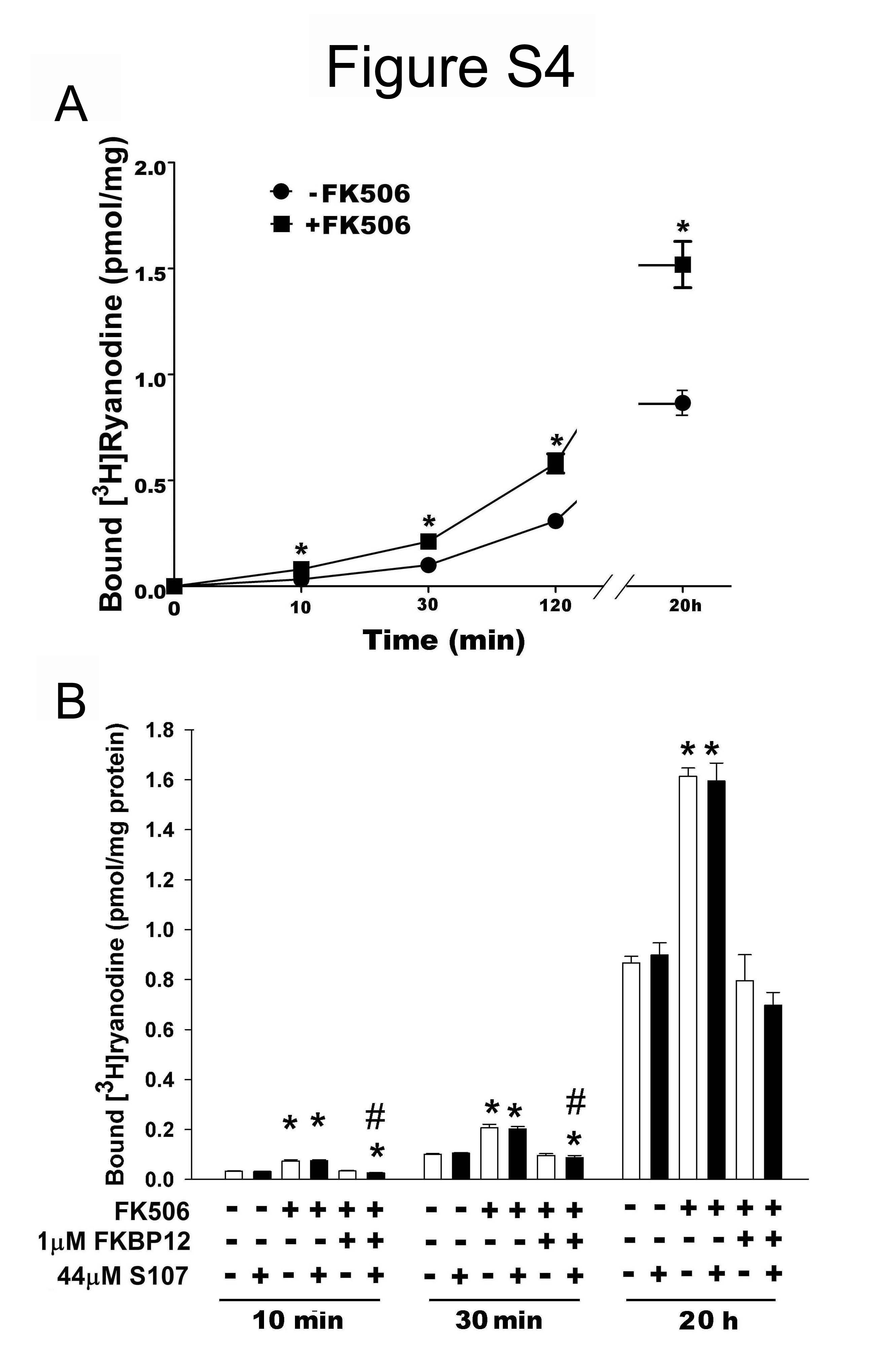

Supplement: Figure S4 — Effects of FKBP12 and S107 on [3H]ryanodine binding to SR vesicles not treated and treated with FK506. (A) Time course of specific [3H]ryanodine binding. Vesicles treated and not treated with FK506 were incubated at 24°C for indicated times in 0.25 M KCl, 20 mM imidazole, pH 7.0, 3 nM [3H]ryanodine, 7 µM free Ca2+, 5 mM GSH and protease inhibitors. Removal of FKBP12 increased RyR1 activity, as indicated by an increase in [3H]ryanodine binding. Data are the mean ± SD of 3–4 experiments. *p<0.05 compared to vesicles minus FK506. (B) Effects of FKBP12 and S107 on [3H]ryanodine binding. SR vesicles treated and not treated with FK506 were incubated at 24°C for the indicated times in the above buffer containing the indicated concentrations of FKBP12 and S107. Data show that in the absence of added FKBP12, S107 did not alter [3H]ryanodine binding to SR vesicles with (−FK506) or freed (+FK506) of FKBP12, whereas S107 significantly decreased [3H]ryanodine binding to FKBP12-depleted vesicles in the presence of FKBP12 after preincubation for 10 min and 30 min. Data are the mean ± SEM of 4–6 experiments. *p<0.05 compared to vesicles minus FK506 at 10 and 30 min, respectively. #p<0.05 compared to FKBP12-depleated vesicles plus added FKBP12 and minus S107 at 10 and 30 min, respectively. (TIF) [file pone.0054208.s004.tif]

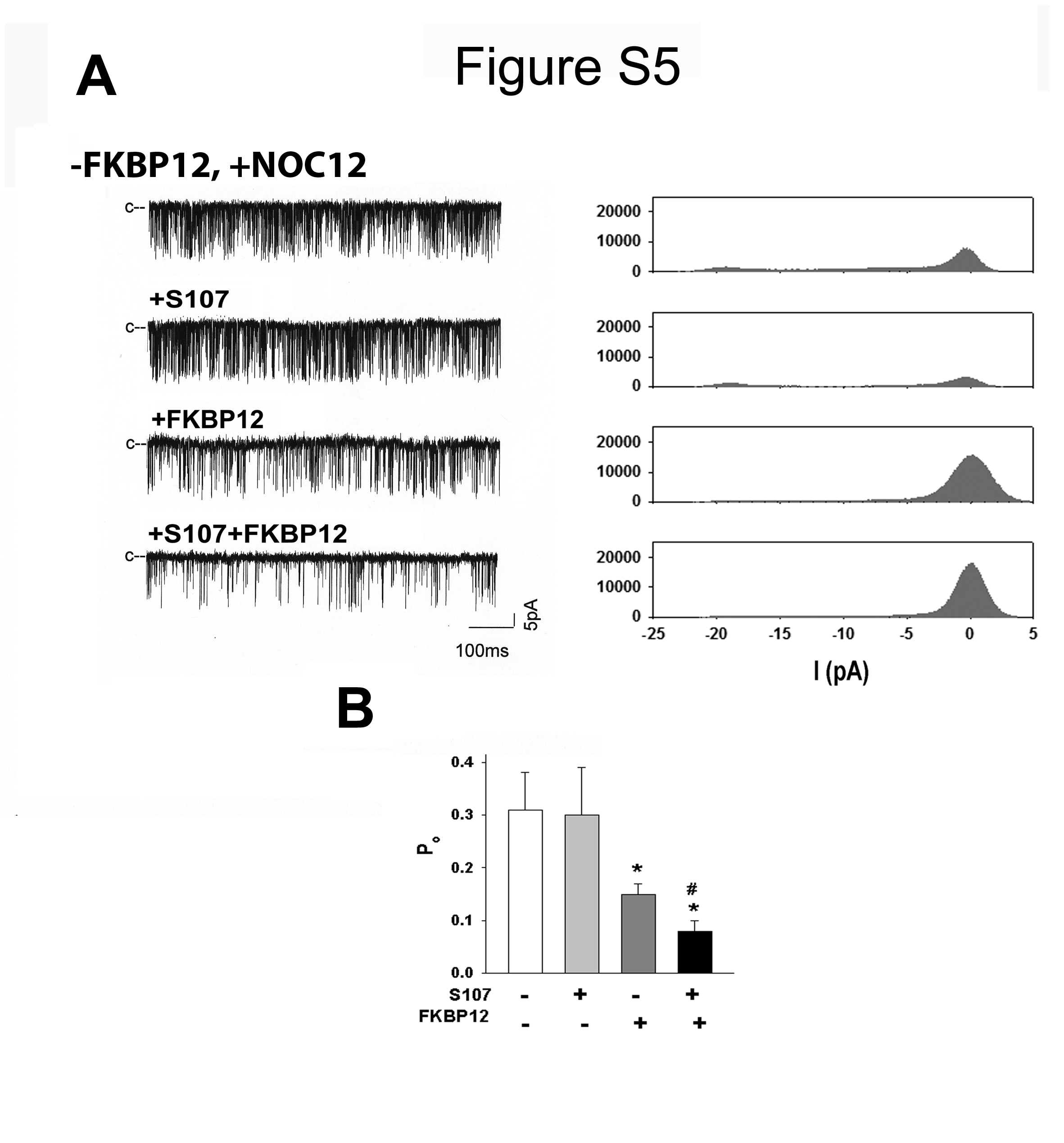

Supplement: Figure S5 — Single channel measurements with NOC12-treated RyR1s. (A) FK506-treated SR vesicles were incubated for 30 min at 24°C with 0.10 mM NOC12 in 0.3 M sucrose, 0.25 M KCl, 20 mM imidazole, pH 7.0 without (top trace), with 44 µMS107 (trace 2), 5 µM FKBP12 (trace 3) or 44 µM S107 plus 5 µM FKBP12 (bottom trace). Vesicles were then fused to a lipid bilayer and recorded at 2 µM cis cytoplasmic Ca2+ and −35 mV as described in Materials and Methods. Representative single channel recordings (downward deflections from closed levels, c–) (left) and current histograms (right) are shown. (B) Single channel data were obtained as described in A. Mean channel open probabilities (Po) were greater in the presence of NOC12 (this figure) than absence of NOC12 (Fig. 8) by 11% in absence of S107 and FKBP12, by 11% in presence of S107, by 3% in presence of FKBP12, and 35% in presence of S107 and FKBP12. Data are the mean ± SEM of 5–8 single channel recordings. *p<0.05 compared to RyR1s not incubated with S107 and FKBP12, #p<0.05 compared to RyR1s incubated with FKBP12 in absence of S107. Significance of differences of data was analyzed with Student’s t-test. (TIF) [file pone.0054208.s005.tif]
